# Rapamycin and Minocycline Treatment Does Not Rescue Behavioral and Molecular Changes Induced by Early-Life Seizures in Female Mice

**DOI:** 10.3390/neurosci7030055

**Published:** 2026-05-05

**Authors:** Sydney F. Pell, Katherine J. Blandin, Taylor R. Bradish, Chloe V. Lau, Danielle Santana-Coelho, Madison Wallis, Colton W. Kelley, Josh J. Thayil, Ashley Smelley, Gautham Cheliah, David A. Narvaiz, Kendall N. Lally, Leighton Douglas, Joaquin N. Lugo

**Affiliations:** 1 Department of Psychology and Neuroscience, Baylor University, Waco, TX 76798, USAkatie_blandin1@baylor.edu (K.J.B.); chloe_lau1@baylor.edu (C.V.L.); madisonawallis@gmail.com (M.W.); ckelley@ucdavis.edu (C.W.K.); josh_thayil1@baylor.edu (J.J.T.); ashley.smelley@yahoo.com (A.S.); david.narvaiz@utsa.edu (D.A.N.); kendallnicole002@gmail.com (K.N.L.); ldoug0309@gmail.com (L.D.); 2 Long School of Medicine, The University of Texas at San Antonio, San Antonio, TX 78229, USA; coelhod@uthscsa.edu; 3 Department of Biology, Baylor University, Waco, TX 76798, USA; 4 Baylor Center for Developmental Disabilities, Baylor University, Waco, TX 76798, USA

**Keywords:** epilepsy, autism, neuroinflammation, mTOR, neonatal, early life, intracellular signaling, autism, comorbidity

## Abstract

Early-life seizures lead to long-term behavioral deficits, stimulate cytokine release, and disrupt the intracellular PI3K/AKT/mTOR signaling pathway. This study examined whether inhibiting the mTOR pathway, neuroinflammatory signaling, or both reduces behavioral comorbidities in adulthood. Female C57BL/6J mice received kainic acid on postnatal day 10 to induce status epilepticus. Three hours later, the mice were treated with saline, minocycline, rapamycin, or both. Three months later, behavioral assessments were conducted that measured activity, anxiety, social behavior, repetitive behavior, and learning. Early-life seizures resulted in social behavior deficits in the social chamber test, altered anxiety in the elevated plus maze, and an increase in repetitive behavior in the nose poke assay. Rapamycin and minocycline/rapamycin groups showed reduced distance traveled in the saline groups. We did not find any changes in cytokines IL6, IL-1β, and TNFα in the hippocampus or cortex using RT-qPCR. Through Western blotting, we found that rapamycin reduced the phosphorylated S6 levels. Minocycline decreased phosphorylated S6 in controls, but restored phosphorylated S6 levels in the seizure group. Early-life seizures had long-term impacts on behavioral comorbidities. Rapamycin and minocycline, alone or combined, did not restore the behavioral or molecular changes after early-life seizures. These findings clarify the behavioral outcomes after early-life seizures and therapeutic modulation.

## 1. Introduction

Epilepsy is a chronic neurological disorder characterized by abnormal electrical brain activity and recurrent, spontaneous epileptic seizures [1,2]. It affects about one percent of the population, and between 4 and 10% of children experience at least one seizure during childhood [3]. Convulsive disorders are the most common neurological conditions in children, with the highest incidence of epilepsy occurring during the first year of life [3,4]. Neonatal seizures, which occur within the first four weeks of life, and particularly those in the first week, are associated with poor prognosis and adverse neurological outcomes [4].

Epilepsy is frequently accompanied by a range of comorbidities, among which is autism spectrum disorder (ASD). ASD is a neurodevelopmental disorder characterized by abnormalities in communication and social interactions, and restrictive and repetitive behaviors and interests [1]. In the general population, ASD affects about 1 in 31 children, or between 1 and 3% of the worldwide population [5]. However, in children diagnosed with epilepsy, about 30% of them also meet the diagnostic criteria for ASD. Conversely, 5–46% of children with ASD also have, or later develop, epilepsy [2,6]. In a population-based study, Jokiranta et al. (2014) reported that childhood autism showed a nearly tenfold increase in odds for all epilepsy syndromes [1]. The association was especially high for females with ASD and intellectual disabilities. Although many studies have demonstrated a strong association between autism and epilepsy, the mechanisms underlying this relationship remain poorly understood.

One proposed mechanism that could underlie the ASD comorbidity in epilepsy is the mammalian target of rapamycin (mTOR) pathway. The mTOR kinase is a serine/threonine protein kinase that plays a central role in many cellular processes, including cell growth and proliferation, autophagy, and cytoskeleton organization [7,8]. In the brain, mTOR regulates the synaptic plasticity, learning, and neurogenesis [9]. This pathway is activated by several signals, including hormones and trophic factors, intracellular adenosine monophosphate levels, and PI3K/Akt kinases [10]. The mTOR signaling pathway consists of two functionally distinct complexes: mTOR complex 1 (mTORC1), which is sensitive to rapamycin inhibition and regulates protein and lipid synthesis and autophagy, and mTOR complex 2 (mTORC2), which is rapamycin-insensitive and regulates cytoskeletal organization, dendritic growth, and cell survival [8,9]. The mTOR pathway also contributes to immune regulation, influencing both innate and adaptive immune responses [7].

Dysregulation of the mTOR pathway has been implicated in both genetic and acquired epilepsy syndromes, as well as ASD. Fragile X Syndrome (FXS), phosphatase and tensin homolog (PTEN) mutations, and the Tuberous Sclerosis Complex (TSC) all exhibit mTOR hyperactivation and are associated with high rates of both epilepsy and ASD [9,11]. The mTOR pathway has been shown to become hyperactive after multiple types of experimentally induced status epilepticus (SE) [12]. In rats, it has been found that the induction of SE via pilocarpine or kainic acid activates the mTOR pathway in the brain within 24 h after seizure activity, with sustained activation several days later in the hippocampus [13,14]. Our laboratory has previously shown that repeated flurothyl-induced seizures in C57BL/6J mice result in mTOR hyperactivity [15].

Chronic inflammation contributes to epileptogenesis, and pre-existing neuroinflammation increases susceptibility to seizures [16]. Neuroinflammation and seizures are in a dysregulated positive feedback loop such that seizures induce neuroinflammation, which increases the propensity for recurrent seizures and thus perpetuates chronic inflammation. Neuroinflammatory processes have likewise been linked to ASD, with increased levels of interleukin-1β (IL-1β), interleukin-6 (IL-6), and interleukin-8 (IL-8) in the brain, cerebrospinal fluid, and peripheral blood of individuals with ASD [17]. However, the interaction between mTOR signaling and neuroinflammation after seizures, and the contribution of these processes to long-term neurological and behavioral outcomes, remains unclear. Elucidating these mechanisms may identify novel therapeutic targets for both epilepsy and ASD. The 3 mg/kg dose used in this study successfully inhibited mTOR activation in neonatal male rats that received hypoxia on postnatal day 10 [18]. The literature on the dose of minocycline is more variable. A dose of 20 mg/kg 3 h after status epilepticus on postnatal day 35 mice (sex not specified) reduced microglia activation [19]. A more recent study used a 50 mg/kg dose of minocycline over 2 days and found that it reduces hypoxia-induced seizures in 7-week-old male mice [20]. It is important to note that these studies were not conducted in females, and a range of doses for minocycline were used.

In the present study, we aimed to investigate whether a single episode of SE in early life induces ASD-like behaviors in adulthood and whether treatment with minocycline to reduce inflammation, rapamycin to inhibit mTOR signaling, or their combination can rescue these behavioral phenotypes in female mice. Sex hormones such as estrogen and progesterone have well-established neuroprotective and anti-inflammatory roles and can modulate both mTOR signaling and microglial activation. These factors may alter the baseline neurobiological responses and influence pharmacological efficacy [21,22]. Adult female mice were evaluated using a comprehensive behavioral battery assessing locomotion, exploratory activity, anxiety-like behavior, sociability, repetitive behaviors, and fear-based memory. We also collected acute tissue one day post-seizures to measure the cytokine levels in the hippocampus and cortex, as well as mTOR activity in the hippocampus. We hypothesize that mice that were exposed to early-life SE would display ASD-like behavioral deficits; specifically, reduced sociability, increased repetitive behaviors, hyperactivity, heightened anxiety, and impaired memory. The combined treatment of rapamycin and minocycline is hypothesized to ameliorate these effects. We further hypothesize that SE induced by kainic acid would increase cytokine expression and mTOR hyperactivation.

## 2. Materials and Methods

### 2.1. Animals

For the experiments in this study, we used C57BL/6J female mice (The Jackson Laboratory, Bar Harbor, ME, USA), which were bred and group-housed with their siblings in standard housing cages at Baylor University. The mice were housed and tested according to the guidelines by Baylor University’s Institutional Care and Use Committee and the National Institute of Health’s Guide for the Care and Use of Laboratory Animals. The mice were weaned at about three weeks of age. The mice were housed in a temperature-controlled colony room maintained at 22 °C and were kept on a 12 h light, 12 h dark diurnal cycle with ad libitum access to food and water.

### 2.2. Seizure Induction

On postnatal day (PD) 10, the mouse pups were weighed, marked, and allowed to habituate to the testing room for 30 min in their home cages. The pups were sorted randomly into either the control or kainic acid (KA) group. KA pups were intraperitoneally injected with 2.5 mg/kg KA at a dosage of 0.5 mg/mL (Biotechne Tocris, USA catalog number 7065, Minneapolis, MN, USA). The control pups went through a sham procedure using vehicle injections of 0.9% physiological saline to the same dosage specifications as the KA pups. After the injections, the mice were placed individually in plastic containers (6 ¼ × 4 ¼ × 5 5/8) with clean bedding on top of a warmed heating pad (~35 °C) to maintain their body temperature. The seizure severity was measured using a modified Racine scale [23]. The mice were monitored and video-recorded throughout their status epilepticus (SE) episode, which began at about 30 min post-injection, for a length of 2 h. SE activity was monitored as continual tonic–clonic behavior. We also measured the forelimb motion and hindlimb motion, but the conformation of SE was dependent on continuous tonic–clonic behavior for more than 10 min. Only mice that had SE were included in the study. Once the visible SE activity stopped, all pups were administered a subcutaneous injection of 0.5 mL of 0.9% physiological saline to counter the dehydration effects of seizures. The mice were left in their containers for another hour, and then treatment was administered.

### 2.3. Treatment

Rapamycin (LC Laboratories, Woburn, MA, USA) was dissolved in a vehicle solution containing 4% ethanol, 5% polyethylene glycol 400 (Sigma, St. Louis, MO, USA), and 5% Tween 80 (Sigma). The final concentration of rapamycin was 0.75 mg/mL and administered at a dose of 3 mg/kg. Minocycline was dissolved in saline for a concentration of 12.5 mg/mL and administered at a dose of 50 mg/kg. Three hours post-injection of KA, C57BL/6J mice were randomly assigned to one of four treatments. This time point was used because early molecular cascades, such as mTOR activation and neuroinflammatory signaling, can occur rapidly—from within minutes to 1 h following seizures [13,16]. All mice received two intraperitoneal (i.p.) injections of a combination of 0.9% physiological saline, rapamycin, or minocycline. The volume injected for seizure induction (saline or KA) and treatment (minocycline, rapamycin, saline) was the same for each individual pup. The treatment groups were saline/saline, saline/minocycline, saline/rapamycin, and minocycline/rapamycin. With the combined administration of both minocycline and rapamycin, the drug that was administered first alternated between each mouse to counterbalance the injection location. Following the administration of the treatments, pups were left in their containers on a warmed heating pad (~35 °C) and monitored for 15 min before they were toe clipped for later identification. The pups were then returned to their home cages and allowed to grow up for behavioral testing in adulthood.

### 2.4. Behavioral Testing

All behavioral tests were conducted during the light phase between 8:00 am and 5:00 pm. Testing began at about two months of age and lasted for four weeks. No more than two behavioral tests occurred per week, allowing at least a two-day inter-test interval, and they were ordered from least invasive to most invasive in the following order: Open Field, Elevated Plus Maze, Nose Poke, Social Chamber, Marble Burying, and Delayed Fear Conditioning [24]. However, the role of testing could alter later behavior. All testing areas were cleaned with 30% isopropyl alcohol between each testing session. For all behavioral testing, mice were weighed, and their tails were marked with a black Sharpie permanent marker for identification. They were then allowed to habituate in the room where the test would occur for a period of 30 min. Mice that were tested for open field, elevated plus maze, nose poke, and social chamber were tested in the same room as the mice that were being acclimated. We are able to simultaneously test 12–15 mice at one time in the marble burying test, so all mice were tested at the same time. The mice that were acclimated in one room were tested for delayed fear conditioning in another room. This way, their fear response could not be heard by the other testing mice. The beginning sample size of all groups was as follows: N Total = 81 (control, saline = 10; control, minocycline = 11; control, rapamycin = 11; control, minocycline, rapamycin = 10; KA, saline = 9; KA, minocycline = 10; KA, rapamycin = 10; and KA, minocycline, rapamycin = 10).

### 2.5. Open Field

To investigate changes to locomotion, stereotyped, and anxiety-like behaviors, all mice underwent the open field test (OF) [25]. After habituation, the mice were placed individually in the center of an acrylic arena (41 cm × 41 cm × 32 cm) with a white base in the testing room. The experimenter left the room, and the mice were allowed to explore the arena for 30 min. Behavioral activity within the chamber was detected and stored using Fusion Software (version 4.5; Omnitech, Columbus, OH, USA). Activity was recorded when the mouse repeatedly broke the same beams within the apparatus’s field. The total distance traveled, rest time, rearing frequency, and rearing time were analyzed as exploratory and locomotor behaviors. Circling behavior and grooming determined differences in stereotyped behavior. Distance traveled and time spent in the center of the open field (the inner 50% of the chamber) were compared to evaluate anxiety-like and exploratory behavior. After each trial, the mice were removed from the arena and placed in a holding cage (30 cm × 18 cm × 13 cm) with bedding until all animals from the home cage were tested, at which time all the mice were placed back in their home cage.

### 2.6. Elevated Plus Maze

The elevated plus maze (EPM) was used to further evaluate the presence of anxiety-like behaviors [26,27]. A plus-shaped maze was elevated 40 cm above the ground. Two opposing arms were enclosed with acrylic walls (15 cm tall), and the other two opposing arms were open. Each arm was 30 cm × 5 cm, and the central platform was 5 cm × 5 cm. Following habituation, mice were placed onto the central platform facing an open arm. They were allowed to explore the maze for 10 min without an experimenter present. Ethovision XT video tracking software version 8 (Noldus, Wageningen, The Netherlands) was used to measure activity in the maze. The total distance traveled and velocity were analyzed as measures of locomotor and exploratory activity. Time spent in the open and closed arms, as well as the number of entries into the arms, were compared to evaluate anxiety-like behavior. After completion of the elevated plus maze test, the mice were housed in a holding cage until all mice from that home cage were run.

### 2.7. Nose Poke

To measure repetitive behavior, mice were run through a nose poke (NP) test [28,29]. After habituation, mice were individually placed in the center of an acrylic chamber (41 cm × 41 cm × 32 cm) containing a 1.9 cm floorboard perforated with 16 holes. Each hole was 2.54 cm in diameter. The mice were allowed to explore the chamber for 10 min, and their nose pokes were live-scored. A nose poke was counted when a mouse entered its head into a hole on the floorboard to eye level. Total nose pokes, latency to first poke, location of hole poked (center and outside), and number of repeated hole entries were recorded and analyzed. After finishing the task, the mice were transferred to a holding cage until all mice in the home cage had been run.

### 2.8. Three-Chamber Social Task

To evaluate their social preference, mice were run through a three-chamber social test (SC) [30]. The testing apparatus was a clear acrylic box (24 in. × 16 in. × 9 in.) with three chambers (8 in. × 16 in. × 9 in.) separated by a clear acrylic wall and removable partitions. Testing occurred during two phases: the baseline phase and the testing phase. For each phase, a wire cup (3.25 in. diameter × 4 in. tall) was placed inverted in each of the side chambers with a 32-ounce plastic bottle (10.125 in. tall) filled with water placed above the cup to prevent the mice from climbing or moving the cups. A novel mouse was placed in the cup for one hour for two days prior to testing to be habituated to the cup. During the baseline phase, the two wire cups were empty. The mice were placed in the center chamber with the partitions removed and were allowed to freely explore the chambers for 10 min. During the testing phase, an age- and sex-matched novel mouse was placed in one of the inverted cups and a novel object (Lego^®^ block) within the other cup. The mice were placed back in the center chamber with clear acrylic inserts that blocked the entry into each partition. The inserts were then removed, and mice were allowed to freely explore the chambers for 10 min. The novel mouse and object were placed on different sides for each test subject as a counterbalance to prevent side preference. Both phases were video-recorded and scored later by an experimenter that was blind to the group of the mouse. Time spent in each chamber, number of entries into each chamber, time spent at the cups, and number of times interacting with the cups were measured in both phases. The time was measured by the nose of the animal facing the cup and was within approximately 1 cm of the cup. One investigator scored all of the videos and was blinded to the condition of the experimental group. After both phases were completed, the mouse was returned to their home cage, and the partner mice were switched.

### 2.9. Marble Burying

As a measure of stereotypic behaviors, the mice underwent a marble burying (MB) task as used previously [31,32]. After habituation, mice were placed in a plastic cage containing a grid-shaped pattern of 20 black marbles on four centimeters of bedding. The marbles were aligned so that there were 5 rows of 4, and so that there was room to place the mouse in the rear of the cage without disrupting the marbles. Some bedding from the home cage was sprinkled into the test cage to decrease the novelty of the new cage. The mice were placed in the test cage and left for 30 min. After the mice were returned to their home cages, the number of marbles at 50%, 75%, 100%, and the total buried was scored. Marbles that are buried completely under the bedding, but with parts of the marble that can still be seen through the bedding, are called 100% buried. Marbles that are totally buried cannot be seen at all through the bedding.

### 2.10. Delayed Fear Conditioning

To evaluate any effects on contextual and cue-based fear memory, mice underwent a delay fear conditioning (DFC) task [33,34]. Testing occurred over two days and consisted of three trials. On day one, mice went through a conditioning trial where they learned to associate an auditory stimulus (80 dB white noise) and the testing environment with an aversive stimulus (0.7 mA shock for 2 s). The mice were placed individually in a transfer cage (30 cm × 18 cm × 13 cm) with normal bedding and then placed individually in the fear chambers. The conditioning trial consisted of a baseline measurement and the playing of the 80 dB auditory stimulus, followed by a shock (tone 1 and inter-trial interval 1 [ITI1]). The auditory stimulus and shock were repeated one more time. The freezing behavior was measured during each phase (baseline, tone 1, ITI1, tone 2, and ITI2). The mice were placed in a holding cage together until all mice from the home cage were run. The first trial of day two was a contextual test. The mice were placed back in the original chamber for 5 min while the freezing behavior was measured. The mice were then returned to their home cage for two hours, and the chambers were cleaned. The second trial of day two was a cued test. The environment of the chamber was changed to provide a novel context. The changes to the environment include the following: the transfer cages had paper towel bedding in them, different chamber floors and walls were used, the lighting in the chamber was changed, vanilla was placed in the chamber to change its smell, and the chamber fan was turned on. The freezing behavior of the mice was again measured in the new context for 180 s. Finally, the freezing behavior was measured while the mice were presented with the 80 dB white noise for an additional 180 s.

### 2.11. Molecular Testing

Hippocampal and cortical tissue were collected for quantitative reverse transcription–polymerase chain reaction (qRT-PCR) and Western blotting according to pre-established laboratory protocols. On PD11 (24 h post-induction), the mice pups were anesthetized using isoflurane (Covetrus, Dublin, OH, USA) and perfused using 1× PBS to clear the brain of peripheral blood. The mice were then decapitated, and their brains were removed. The hippocampus and a portion of the cortex were dissected from the brain, rinsed in 1× PBS, placed on dry ice, and stored at −80 °C until processed. For qRT-PCR, the beginning sample size was 10 samples per group, and for Western blots, the sample size was 8 mice per group.

### 2.12. RT-qPCR

The hippocampal and cortical expression of cytokines was examined through qRT-PCR, using previously described methods [35]. RNA was isolated using the RNeasy Mini Kit (Qiagen, Hilden, Germany). We examined the cortex dorsal to the hippocampus. RNA was reverse-transcribed into single-stranded complementary DNA according to the manufacturer’s instructions on the High-Capacity cDNA Reverse Transcription Kit (Applied Biosystems, Carlsbad, CA, USA). For the final stage of RT-qPCR, TaqMan primers (actin, interleukin-1beta [IL-1β], interleukin-6 [IL-6], and tumor necrosis factor-alpha [TNF-α]) and the cDNA samples were thawed on ice. The groups were compared to the control saline–saline (control insult and treatment) animals. The relative expression levels of IL-1β, IL-6, and TNF-α were quantified using the comparative threshold (2^−ΔΔCt^) method [36].

### 2.13. Western Blotting

To examine the effect of seizures and the treatments on the mTOR pathway protein expression, Western blotting was performed as previously described [37]. The hippocampal tissue was homogenized in a glass Dounce tissue grinder in ice-cold homogenization buffer containing 0.32 M sucrose, 1 mM EDTA, 5 mM HEPES, and protease inhibitor cocktail (P8340, Sigma, USA). The samples in the total tubes were boiled for 5 min at 100 °C, vortexed, and stored at 4 °C until further processing. The samples were grouped into 8 groups of 8 samples. Westerns were set up to run groups one through four for one week and groups five through eight the next week. The antibodies were grouped to run pS6 (Ser240/244) (catalog number 5364; Cell Signaling, Danvers, MA, USA), S6 (catalog number 2217; Cell Signaling, USA), and actin (A2228; MilliporeSigma, St. Louis, MO, USA) the next week for each set of groups. For analysis, Westerns were desensitized using Alpha View SA version 3.4.0 (R&D Systems, Minneapolis, MN, USA). Using the multiplex band analysis tool, boxes were drawn around all 16 bands (8 from the top membrane and 8 from the bottom membrane) and for a regional background. The protocols were saved and used to analyze the bands on all four images of each gel. The analyses from Alpha View were saved, and the ratio of pAKT to AKT, pS6 to S6, AKT to actin, and S6 to actin were compared between groups to identify differences in the mTOR activity.

### 2.14. Statistical Analysis

Statistical analysis was carried out using SPSS version 29 (IBM, Armonk, NY, USA), and all graphs were created using GraphPad (Prism 7). Two-way repeated measures analysis of variance (ANOVA) was used to investigate the interactions between insult (control, KA) and treatment (saline, minocycline, rapamycin, minocycline and rapamycin). Day one of delayed fear conditioning was analyzed by a three-way repeated measures ANOVA with a within-subjects factor comparison of the trial (baseline, tone 1, inter-trial interval 1, tone 2, inter-trial interval 2). For the analysis of cytokines, the comparative threshold method of quantification was used to determine the relative gene expression levels. All groups were normalized to the control saline–saline group, and two-way ANOVAs were used to assess any differences between groups. For Western blot analysis, two-way ANOVAs were used, and all groups were normalized to the average of the control saline–saline group per blot. The interactions were evaluated using pairwise comparisons with LSD adjustment for multiple comparisons. For significant within-subjects effects, pairwise comparisons were run to find significance within the groups. The level of significance was *p* < 0.05, and data were reported as the mean ± standard error of the mean (SEM). Any animals that were excluded or may have died during testing were reported in the Results Section for that specific behavior.

## 3. Results

### 3.1. Open Field Results

**Figure 1:**
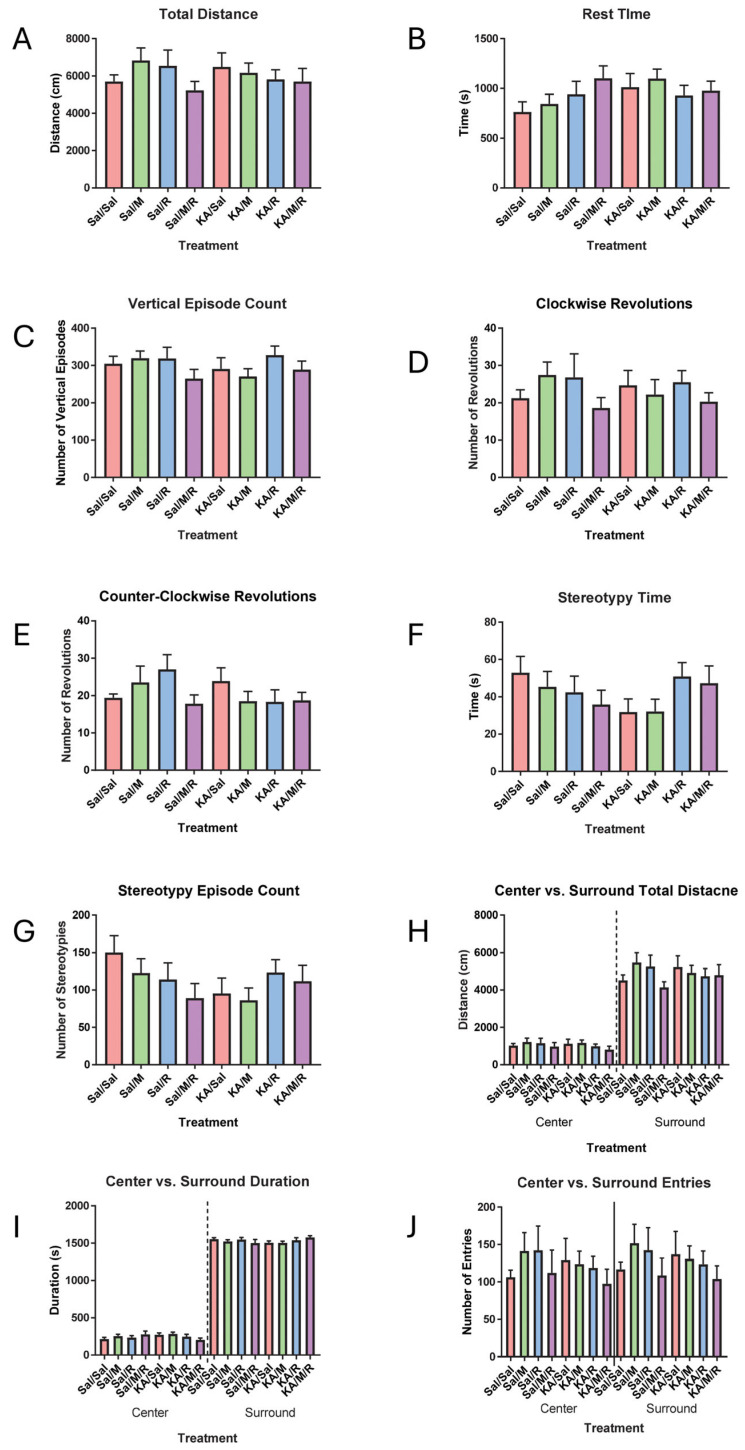
There was no effect of seizures, rapamycin, or minocycline on activity in the open field. Total distance traveled (**A**), amount of time spent resting (**B**), rearing frequency (**C**), circling behavior (**D**,**E**), stereotype time (**F**), or stereotype frequency (**G**). Distance traveled in the center ((**H**) left panel) or surround ((**H**) right panel), time spent in the center ((**I**) left panel) or surround ((**I**) right panel), or mouse visits in the center ((**J**) left panel) or surround ((**J**) right panel). Data are presented as the mean ± SEM. Saline (Sal), kainic acid (KA), minocycline (M), and rapamycin (R). Dotted and solid lines were added to improve clarity in the figure.

Open field was run to assess locomotor and exploratory behavior. A two-way ANOVA of the overall movement found no main effect of insult [F(1,73) = 0.01, *p* = 0.94] or treatment [F(3,73) = 0.94, *p* = 0.43] on the total distance traveled. We did not find an interaction between insult and treatment [F(3,73) = 0.75, *p* = 0.53] (Figure 1A). There was no main effect of insult [F(1,73) = 1.34, *p* = 0.25] or treatment [F(3,73) = 0.64, *p* = 0.59] on the total rest time. There was no interaction between insult and treatment [F(3,73) = 1.44, *p* = 0.24] (Figure 1B). Insult [F(1,73) = 0.19, *p* = 0.67] and treatment [F(3,73) = 1.25, *p* = 0.30] had no effect on the number of vertical episodes and there was no interaction between insult and treatment [F(3,73) = 0.86, *p* = 0.47] (Figure 1C).

To investigate differences in stereotyped behavior, we ran a two-way ANOVA to look at the number of clockwise rotations and found there was no main effect of insult [F(1,73) = 0.02, *p* = 0.90] or treatment [F(3,73) = 1.17, *p* = 0.33]. There was no interaction between treatment and insult [F(3,73) = 0.50, *p* = 0.69] (Figure 1D). Evaluating counterclockwise rotations also found no main effect of insult [F(1,73) = 0.87, *p* = 0.35] or treatment [F(3,73) = 0.71, *p* = 0.55], and no interaction [F(3,73) = 1.73, *p* = 0.17] (Figure 1E). There was no main effect of insult [F(1,73) = 0.39, *p* = 0.53] or treatment [F(3,73) = 0.34, *p* = 0.80] or interaction between treatment and insult [F(3,73) = 1.95, *p* = 0.13] on the stereotypy time (Figure 1F). There was no main effect of insult F(1,73) = 1.08, *p* = 0.30], treatment F(3,73) = 0.57, *p* = 0.64], or interaction between insult and treatment [F(3,73) = 1.64, *p* = 0.19] on the number of stereotypy episodes (Figure 1G).

We also evaluated anxiety-like behavior in the open field by analyzing the total distance traveled and time spent in the center. A two-way ANOVA on the total distance traveled in the center showed no main effect of insult [F(1,73) = 0.27, *p* = 0.60] or treatment [F(3,73) = 0.82, *p* = 0.49]. We did not find an interaction between insult and treatment [F(3,73) = 0.22, *p* = 0.88] (Figure 1H left panel). A two-way ANOVA on the total distance in the surround showed no main effect of insult [F(1,73) = 0.04, *p* = 0.84] or treatment [F(3,73) = 0.82, *p* = 0.49]. We did not find an interaction between insult and treatment [F(3,73) = 1.09, *p* = 0.36] (Figure 1H right panel). Time spent in the center was evaluated using a two-way ANOVA and showed no main effect of insult [F(1,73) = 0.07, *p* = 0.79] or treatment [F(3,73 = 0.52, *p* = 0.67]. There was no interaction between insult and treatment [F(3,73) = 2.06, *p* = 0.11] (Figure 1I left panel). Time spent in the surround area was also evaluated using a two-way ANOVA and showed no main effect of insult [F(1,73) = 0.004, *p* = 0.95] or treatment [F(3,73 = 0.45, *p* = 0.72]. There was no interaction between insult and treatment [F(3,73) = 1.70, *p* = 0.17] (Figure 1I right panel). We also evaluated the number of entries into the center using a two-way ANOVA. We did not find a main effect of insult [F(1,73) = 0.24, *p* = 0.63] or treatment [F(3,73) = 0.59, *p* = 0.63] on the number of entries in the center and surround. We did not find an interaction between insult and treatment [F(3,73) = 0.38, *p* = 0.77] (Figure 1J left panel). We also evaluated the number of entries into the surround using a two-way ANOVA. We did not find a main effect of insult [F(1,73) = 0.14, *p* = 0.71] or treatment [F(3,73) = 0.88, *p* = 0.46] on the number of entries in the center and surround. We did not find an interaction between insult and treatment [F(3,73) = 0.35, *p* = 0.79] (Figure 1J right panel).

### 3.2. Elevated Plus Maze Results

**Figure 2:**
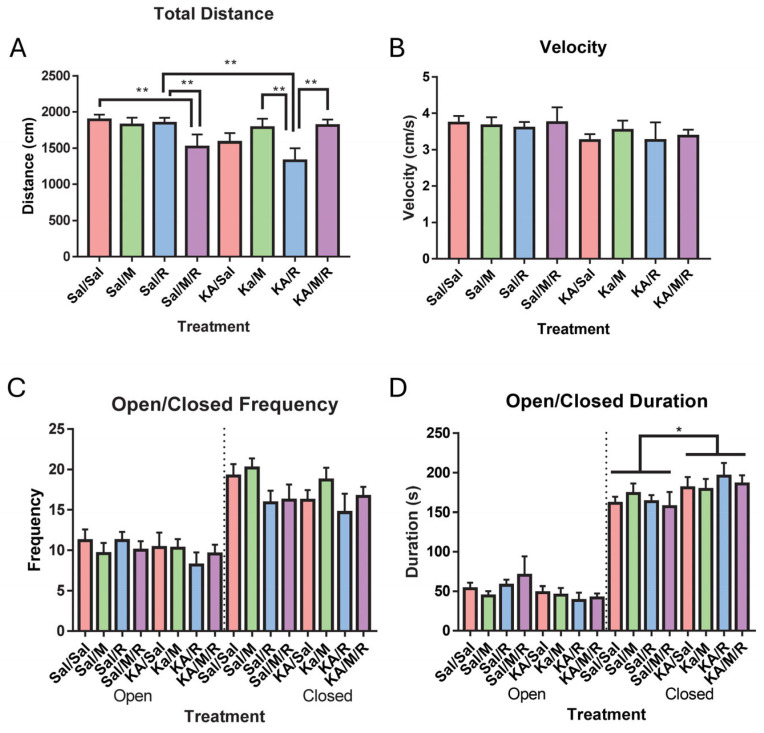
Control minocycline and rapamycin mice traveled less than control rapamycin mice and control saline-treated mice. Total distance in the elevated plus maze test (**A**), velocity traveled (**B**), how often mice entered the open ((**C**) left panel) or closed arms ((**C**) right panel), or time spent in the open arms ((**D**) left panel) and closed arms ((**D**) right panel). Data are presented as the mean ± SEM. * *p* < 0.05 and ** *p* < 0.01. Saline (Sal), kainic acid (KA), minocycline (M), and rapamycin (R). Dotted lines were added to improve clarity in the figure.

The elevated plus maze was used as a secondary measure to assess the locomotor and exploratory behavior. Because of technical errors, two mice had to be excluded from the analysis. The final group sample sizes were as follows: control, saline = 10; control, minocycline = 10; control, rapamycin = 11; control, minocycline, rapamycin = 10; KA, saline = 9; KA, minocycline = 10; KA, rapamycin = 9; and KA, minocycline, rapamycin = 10. We ran a two-way ANOVA on the total distance traveled and did not find a main effect of insult [F(1,71) = 2.91, *p* = 0.09] or treatment [F(3,71) = 1.23, *p* = 0.30]. We did find an interaction between insult and treatment [F(3,71) = 4.43, *p* = 0.01]. Pairwise LSD comparisons showed a significant difference in the rapamycin-treated mice in the KA and control groups, with the control, rapamycin group traveling further than the KA, rapamycin group. Within the KA-induced mice, there was a significant difference in distance traveled between the mice treated with minocycline and rapamycin and with mice treated with both minocycline and rapamycin alone. Within the control mice, there was a significant difference in distance traveled between the mice treated with both minocycline and rapamycin and with rapamycin alone and with mice treated with both minocycline and rapamycin and with saline (Figure 2A). To evaluate the differences in velocity as a measure of locomotor activity, a two-way ANOVA was run and did not find a main effect of insult [F(1,71) = 3.45, *p* = 0.07] or treatment [F(3,71) = 0.18, *p* = 0.91] on the total velocity. We did not find an interaction between insult and treatment [F(3,71) = 0.18, *p* = 0.91] (Figure 2B).

Anxiety-like behavior was also evaluated in the elevated plus maze through two-way ANOVAs of the number of entries into open and closed arms and total time spent in the open and closed arms. Neither insult [F(1,71) = 0.99, *p* = 0.32] nor treatment [F(3,71) = 0.27, *p* = 0.85] had an effect on the total number of entries into the open arms. We did not find an interaction between insult and treatment [F(3,71) = 0.70, *p* = 0.56] (Figure 2C left panel). We did not find a main effect of insult [F(1,71) = 1.39, *p* = 0.24] or treatment [F(3,71) = 2.59, *p* = 0.06] on the total number of entries into the closed arms. There was no interaction between insult and treatment [F(3,71) = 0.40, *p* = 0.75] (Figure 2C right panel). There was no main effect of insult [F(1,71) = 2.68, *p* = 0.11] or treatment [F(3,71) = 0.36, *p* = 0.78] on the total time spent in the open arms. There was no interaction between insult and treatment [F(3,71) = 0.72, *p* = 0.54] (Figure 2D left panel). We did find a main effect of insult [F(1,71) = 5.43, *p* = 0.02] on the total time spent in the closed arms where the KA group spent more time in the closed arms than the control group. We did not find a main effect of treatment [F(3,71) = 0.19, *p* = 0.90] on the total time spent in the closed arms (Figure 2D right panel). We did not find an interaction between insult and treatment [F(3,71) = 0.44, *p* = 0.73]. To assess the within-subject effects of frequency and duration in the open and closed arms, we ran a two-way repeated-measures ANOVA. While we did not find a within-subjects effect on the frequency of entries into the open and closed arms, we did find an effect of insult on time spent in the open and closed arms [F(1,71) = 4.38, *p* = 0.04]. The KA-induced mice spent more time in the closed arms than the saline group (Figure 2D).

### 3.3. Nose Poke Results

**Figure 3:**
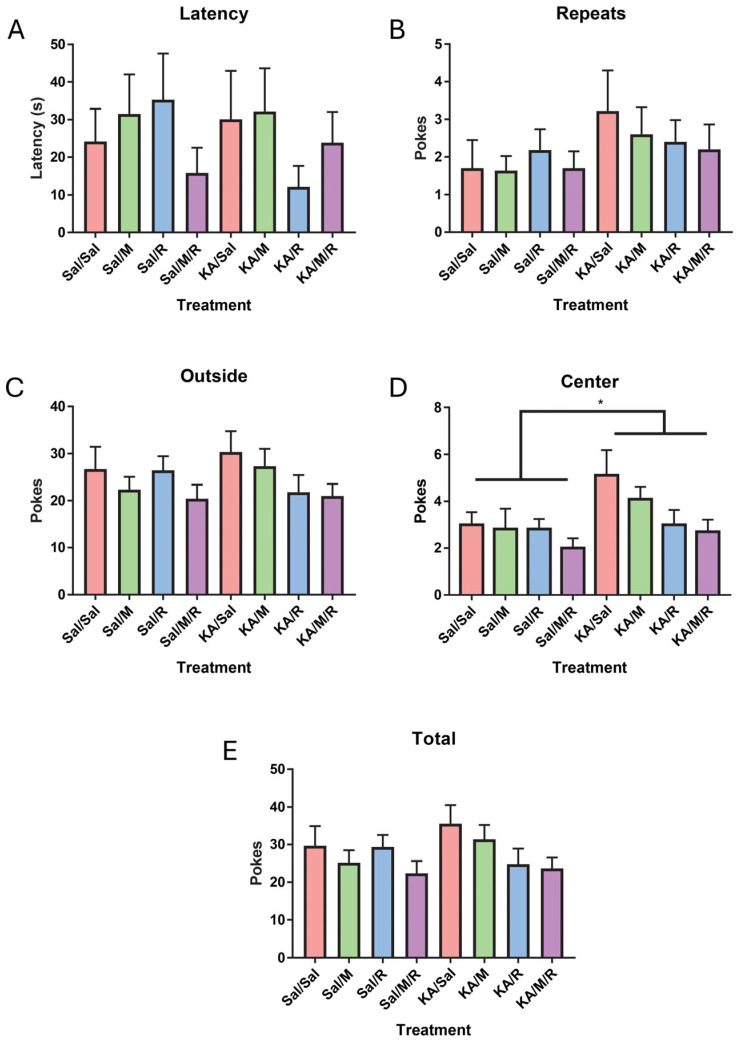
There was no effect of rapamycin or minocycline in the nose poke. Latency to first nose poke (**A**), number of repeated nose pokes (**B**), and nose pokes to outside nose pokes (**C**). Center nose pokes (**D**) and total number of nose pokes (**E**). Data are presented as the mean ± SEM. * *p* < 0.05. Saline (Sal), kainic acid (KA), minocycline (M), and rapamycin (R).

To assess the repetitive behaviors, a nose poke task was run. A two-way AVOVA on latency to first nose poke, number of repeated nose pokes, number of pokes to the outside holes, number of pokes to the center holes, and total nose pokes was used to evaluate these behaviors. We did not find an effect of insult [F(1,73) = 0.09, *p* = 0.77] or treatment [F(3,73) = 0.54, *p* = 0.66] on the latency to first nose poke. There was no interaction between insult and treatment [F(3,73) = 1.07, *p* = 0.37] (Figure 3A). We found no effect of insult [F(1,73) = 2.97, *p* = 0.09] or treatment [F(3,73) = 0.22, *p* = 0.88] on the number of repeated nose pokes. There was no interaction between insult and treatment [F(3,73) = 0.37, *p* = 0.78] (Figure 3B). There were no main effects of insult [F(1,73) = 0.21, *p* = 0.65], treatment [F(3,73) = 1.61, *p* = 0.20], or an interaction between insult and treatment [F(3,73) = 0.76, *p* = 0.52] for nose pokes to outside holes (Figure 3C). A two-way ANOVA on nose pokes to the center holes found a significant main effect of insult [F(1,73) = 5.37, *p* = 0.02] where the KA group had more center pokes than the control saline group. There was no main effect of treatment [F(3,73) = 2.45, *p* = 0.07] on the number of center pokes, and we found no interaction between insult and treatment [F(3,73) = 0.79, *p* = 0.50] (Figure 3D). There was no main effect of insult [F(1,73) = 0.65, *p* = 0.42] or treatment [F(3,73) = 2.00, *p* = 0.12] on total number of nose pokes. There was no interaction [F(3,73) = 0.86, *p* = 0.47] (Figure 3E).

### 3.4. Three-Chamber Social Task Results

**Figure 4:**
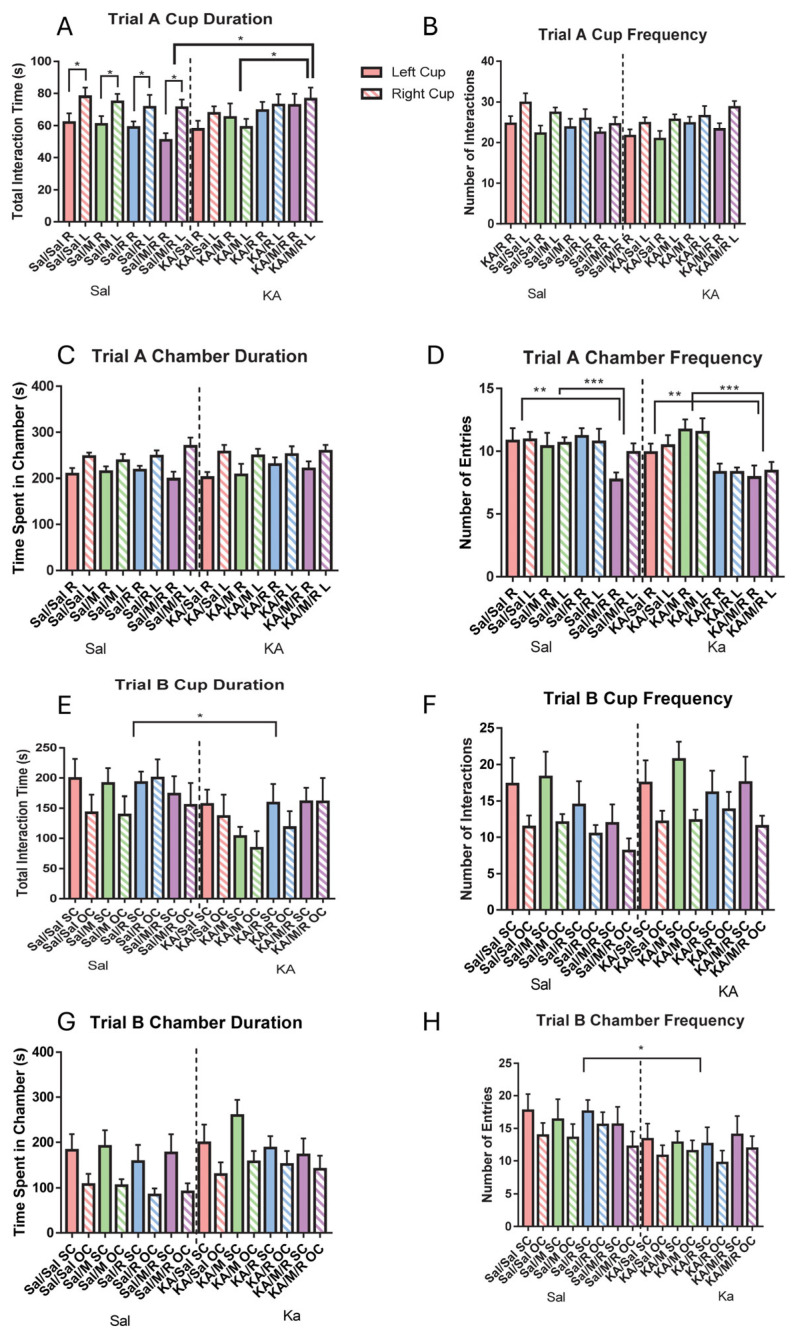
There was no effect of rapamycin or minocycline on social preference. In the baseline phase, measurement of time at the left cup and right cup (**A**). Frequency of interactions at either cup (**B**), the amount of time spent in either chamber (**C**), and frequency in the left chamber and right chamber (**D**). In the testing phase, the duration of interaction at the cup (**E**), and the amount of time spent at either cup (**F**). The duration of time spent at the chamber (**G**) and frequency of interactions in the chamber (**H**). Data are presented as the mean ± SEM. * *p* < 0.05 and ** *p* < 0.01, *** *p* < 0.001. Saline (Sal), kainic acid (KA), minocycline (M), and rapamycin (R). Dotted lines were added to separate the treatment effects and improve clarity.

To investigate social behavior, a three-chamber social task was run, and the data were analyzed. For the baseline phase, a two-way repeated measures ANOVA was run to assess differences in time spent at either cup, frequency of interacting with either cup, time spent in either side chamber, and frequency of entries into the side chambers. We found no main effect of insult [F(1,73) = 0.29, *p* = 0.59] or treatment [F(3,73) = 0.26, *p* = 0.86] on the cup duration. We found a significant interaction between insult and treatment [F(3,73) = 2.76, *p* < 0.05]. Pairwise comparisons show that there was a significant difference in the cup duration between the KA, minocycline and rapamycin group and the control minocycline and rapamycin group. The KA minocycline and rapamycin group spent significantly more time at the cups than the control minocycline and rapamycin group. Within the KA-induced mice, there was a significant difference in the mice treated with minocycline alone and those treated with both minocycline and rapamycin. The minocycline group spent less time at the cups than the mice treated with minocycline and rapamycin. There was also a significant within-subjects effect of insult [F(1,73) = 4.92, *p* = 0.03], where the control mice spent significantly more time at the left cup than at the right cup (Figure 4A). We found no main effect of insult [F(1,73) = 0.05, *p* = 0.82] or treatment [F(3,73) = 0.28, *p* = 0.84] on the frequency of interacting with either cup. There was no interaction [F(3,73) = 2.54, *p* = 0.06] (Figure 4B).

There was no main effect of insult [F(1,73) = 1.33, *p* = 0.25] or treatment [F(3,73) = 1.98, *p* = 0.13] on time spent in either chamber. There was no interaction between insult and treatment [F(3,73) = 0.17, *p* = 0.91] (Figure 4C). We did not find a main effect of insult on the number of entries into either chamber [F(1,73) = 1.97, *p* = 0.17]. We did find a main effect of treatment on the chamber frequency [F(3,73) = 4.80, *p* < 0.01]. Pairwise comparisons showed that the minocycline treatment group entered the chambers more frequently than the minocycline and rapamycin treatment group. The minocycline and rapamycin group entered the chambers less than the saline group. Further univariate two-way ANVOA testing showed that there was no effect of treatment on the number of entries into the left chamber [F(3,73) = 2.69, *p* > 0.05], but there was an effect of treatment on the number of entries into the right chamber [F(3,73) = 5.28, *p* < 0.01]. There was no interaction between insult and treatment on chamber frequency [F(3,73) = 2.31, *p* = 0.08] (Figure 4D).

To assess social preference between a novel sex- and age-matched mouse and a novel object, the testing phase of the task was analyzed by running two-way repeated measures ANOVAs. We found a main effect of insult [F(1,73) = 4.36, *p* = 0.04] on time spent at the two cups. The KA group spent less time at the cups than the control group. Further univariate two-way ANOVA testing showed that the KA group spent less time at the cup that had the mouse than the control group [F(1,73) = 5.98, *p* = 0.02]. There was no significant difference between the KA and control groups in the amount of time spent at the cup with the novel object [F(1,73) = 2.24, *p* = 0.14]. We found no main effect of treatment [F(3,73) = 0.86, *p* = 0.47] and no interaction between insult and treatment [F(3,73) = 0.69, *p* = 0.56] on the time spent at the cups (Figure 4E). We found no main effect of insult [F(1,73) = 2.05, *p* = 0.16] or treatment [F(3,73) = 0.96, *p* = 0.41] on the cup frequency. There was no interaction [F(1,73) = 0.32, *p* = 0.81] (Figure 4F).

There was a marginal effect of insult [F(1,73) = 3.87, *p* > 0.05] but no main effect of treatment [F(3,73) = 0.70, *p* = 0.55] on the chamber duration and no interaction between insult and treatment [F(3,73) = 0.28, *p* = 0.84] (Figure 4G). We did find a main effect of insult on the chamber frequency [F(1,73) = 4.73, *p* = 0.03], with the KA group entering the chambers less frequently than the control group. Further univariate two-way ANOVA testing showed that the KA group went into the chamber with the mouse less often than the control group [F(1,73) = 4.11, *p* < 0.05]. The KA group also went into the chamber with the novel object less than the control group [F(1,73) = 4.17, *p* < 0.05] We found no main effect of treatment [F(3,73) = 0.03, *p* = 0.99] and no interaction between insult and treatment [F(3,73) = 0.40, *p* = 0.76] on the chamber frequency (Figure 4H).

### 3.5. Marble Burying Results

**Figure 5:**
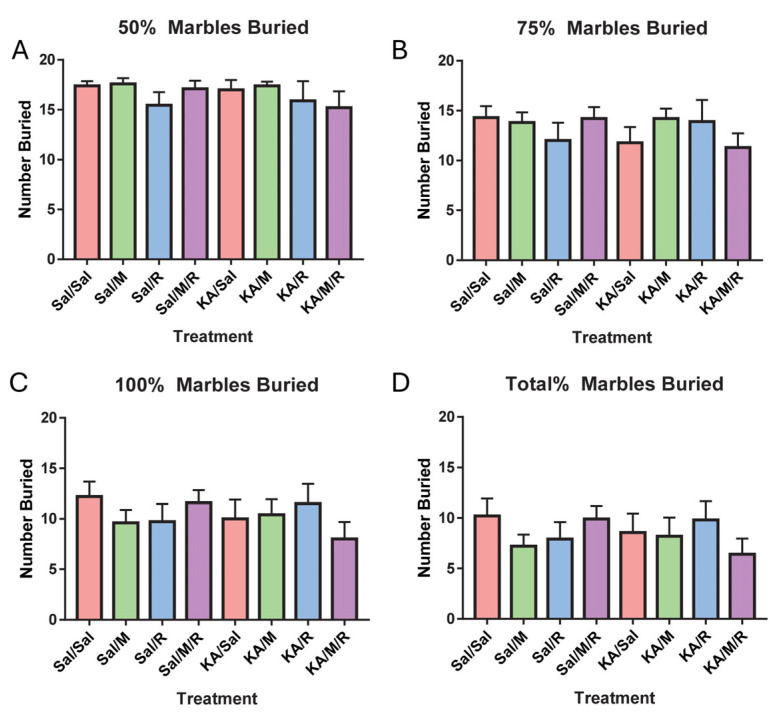
There was no effect of seizures, rapamycin, or minocycline on marble burying. The number of 50% buried marbles (**A**), 75% buried marbles (**B**), 100% buried marbles (**C**), and totally buried marbles (**D**). Data are presented as the mean ± SEM. Saline (Sal), kainic acid (KA), minocycline (M), and rapamycin (R).

As a second assessment of repetitive behavior, we ran the marble burying task. Because of technical errors, one mouse was excluded from data analysis. The final group sample sizes were control, saline = 10; control, minocycline = 10; control, rapamycin = 11; control, minocycline, rapamycin = 10; KA, saline = 9; KA, minocycline = 10; KA, rapamycin = 10; KA, minocycline, rapamycin = 10. A two-way ANOVA on the number of marbles that were 50% buried showed no effect of insult [F(1,72) = 0.38, *p* = 0.54] or treatment [F(3,72) = 1.09, *p* = 0.36]. There was no interaction between insult and treatment [F(3,72) = 0.36, *p* = 0.78] (Figure 5A). We found no main effect of insult [F(1,72) = 0.56, *p* = 0.46] or treatment [F(3,72) = 0.29, *p* = 0.84] on the number of marbles buried 75%, and there was no interaction [F(3,72) = 1.25, *p* = 0.30] (Figure 5B). There was no main effect of insult [F(1,72) = 0.49, *p* = 0.49] or treatment [F(3,72) = 0.26, *p* = 0.86] on marbles that were 100% buried. We did not find an interaction between insult and treatment [F(3,72) = 1.21, *p* = 0.31] (Figure 5C). There was no main effect of insult [F(1,72) = 0.24, *p* = 0.63], treatment [F(3,72) = 0.40, *p* = 0.75], or interaction between insult or treatment [F(3,72) = 1.14, *p* = 0.34] for marbles that were totally buried (Figure 5D).

### 3.6. Delayed Fear Conditioning Results

**Figure 6:**
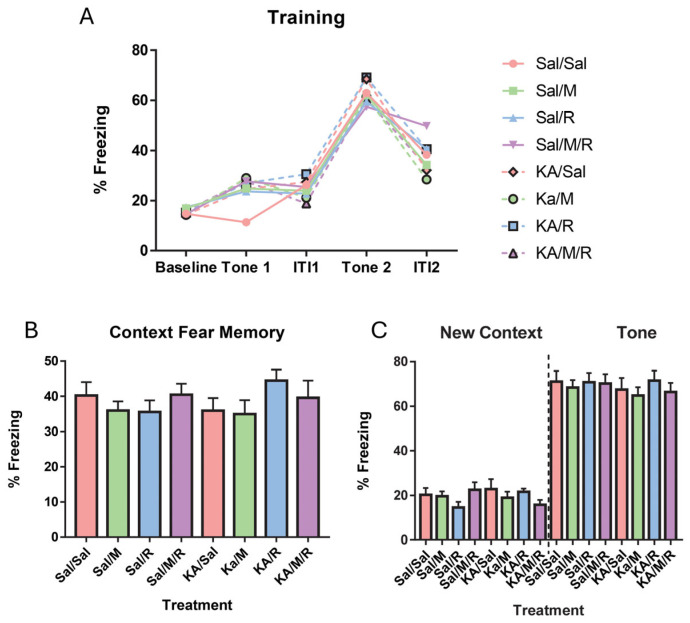
There was no effect of seizures, rapamycin, or minocycline on delayed fear conditioning. Freezing behavior during training (**A**), the first context test (**B**), in the new context (**C**): left of dashed line) or tone tests (**C**): right of dashed line). The dashed line in C was added to separate the effects of the New Context and Tone on the freezing levels of the groups. Data are presented as mean ± SEM. Saline (Sal), kainic acid (KA), minocycline (M), and rapamycin (R).

To assess changes in hippocampal and amygdala-based fear memories, the delayed fear conditioning task was assessed. A two-way repeated measures ANOVA with a within-subjects factor comparison of the trial (baseline, tone 1, inter-trial interval 1, tone 2, inter-trial interval 2) showed that there was no main effect of insult [F(1,73) = 0.02, *p* = 0.88] or treatment [F(3,73) = 0.32, *p* = 0.81] on freezing behavior for day 1. There was no interaction between insult and treatment [F(3,73) = 0.52, *p* = 0.67] (Figure 6A). We found a significant within-subjects effect of insult on training [F(4,292) = 2.73, *p* = 0.03]. In both the KA-induced and controlled groups, there was a significant difference in freezing behavior between all the trials except between tone 1 and ITI 1 (Figure 6A). A two-way ANOVA on the freezing behavior for the context condition on day two showed no significant main effect of insult [F(1,73) = 0.08, *p* = 0.79] or treatment [F(3,73) = 0.74, *p* = 0.53]. There was no interaction found [F(3,73) = 1.28, *p* = 0.29] (Figure 6B). Trial two on day two had two components: a new context and tone cued test. We found no main effect of insult [F(1,73) = 0.08, *p* = 0.79] or treatment [F(3,73) = 0.54, *p* = 0.66] on the freezing behavior for the new context phase. There was no interaction between insult and treatment [F(3,73) = 2.24, *p* = 0.09] (Figure 6C). There was no main effect of insult [F(1,73) = 0.73, *p* = 0.40] or treatment [F(3,73) = 0.42, *p* = 0.74] on the freezing behavior for the tone-cued part of the trial. We found no interaction between insult and treatment [F(3,73) = 0.14, *p* = 0.94] (Figure 6C). A two-way repeated measures ANOVA found no effects of insult [F(1,73) = 0.21, *p* = 0.65], treatment [F(3,73) = 0.23, *p* = 0.87], or an interaction between insult and treatment [F(3,73) = 0.75, *p* = 0.52] on the freezing behavior in the tone test compared to the new context phase (Figure 6C).

### 3.7. RT-qPCR Results

**Figure 7:**
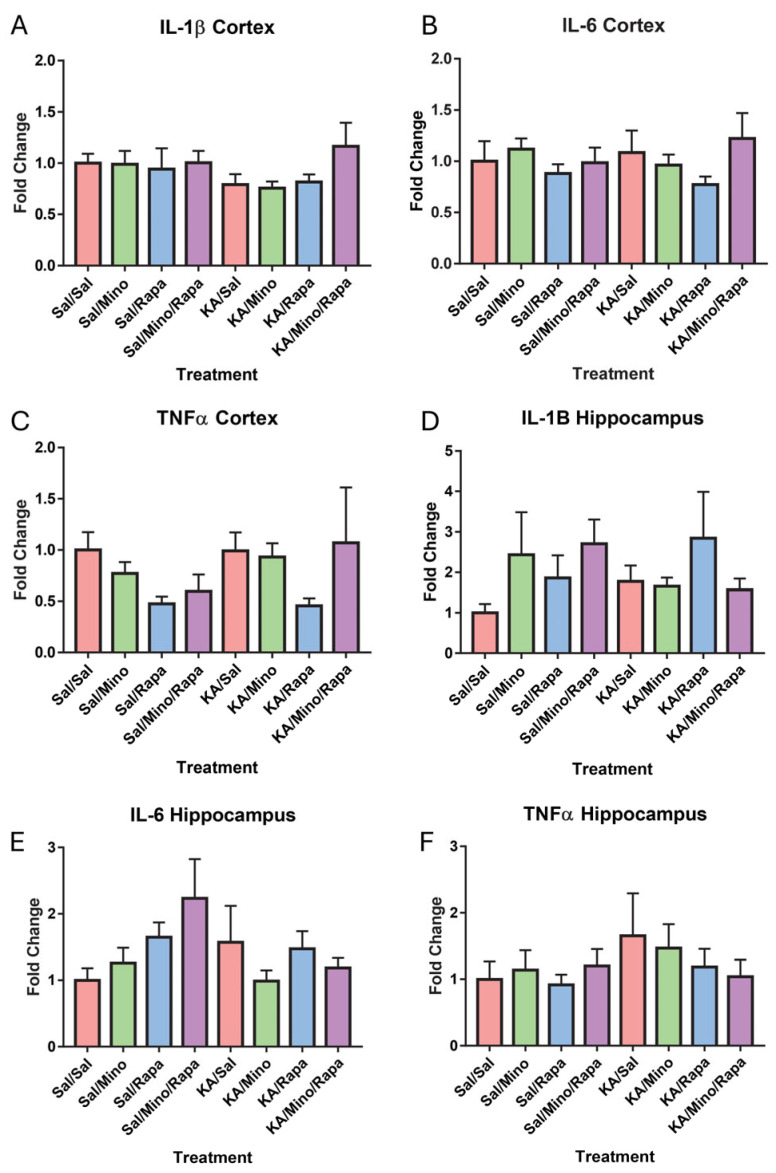
There was no effect of seizures, rapamycin, or minocycline on inflammation markers in the cortex or hippocampus. Inflammatory markers of IL-1β (**A**), IL-6 (**B**), or TNF-α in the cortex (**C**). Inflammatory markers of IL-1β (**D**), IL-6 (**E**), or TNF-α in the hippocampus (**F**). Data are presented as the mean ± SEM. Saline (Sal), kainic acid (KA), minocycline (M), and rapamycin (R).

To assess the changes in inflammation, cortex tissue was run through RT-qPCR to look at expression levels of IL-1β, IL-6, and TNF-α in the hippocampus and cortex. Because of outliers and experimenter error, three samples were excluded from analysis. Final group sample sizes were as follows: control, saline = 10; control, minocycline = 9; control, rapamycin = 10; control, minocycline, rapamycin = 10; KA, saline = 10; KA, minocycline = 9; KA, rapamycin = 9; and KA, minocycline, rapamycin = 10. Two-way ANOVAs were run on the comparative threshold (2^−ΔΔCt^) fold change analysis. We did not find an effect of insult [F(1,69) = 0.86, *p* = 0.36] or treatment [F(3,69) = 1.15, *p* = 0.34] on IL-1β expression levels. We did not find an interaction between insult and treatment [F(3,69) = 0.78, *p* = 0.51] (Figure 7A). We did not find an effect of insult [F(1,69) = 0.02, *p* = 0.90] or treatment [F(3,69) = 1.10, *p* = 0.36] on IL-6 expression levels. We did not find an interaction between insult and treatment [F(3,69) = 0.62, *p* = 0.61] (Figure 7B). Finally, we did not find an effect of insult [F(1,69) = 0.82, *p* = 0.37] or treatment [F(3,69) = 1.82, *p* = 0.15] on the TNFα expression levels. We did not find an interaction between insult and treatment [F(3,69) = 0.48, *p* = 0.70] (Figure 7C).

Additionally, we looked at the inflammation and expression levels of IL-1β, IL-6, and TNF-α in the hippocampus. Because of outliers and experimenter error, four samples were excluded from the analysis. The final group sample sizes were as follows: control, saline = 10; control, minocycline = 10; control, rapamycin = 10; control, minocycline, rapamycin = 9; KA, saline = 10; KA, minocycline = 8; KA, rapamycin = 10; KA, minocycline, rapamycin = 9. A two-way ANOVA on the expression levels of IL-1β did not show an effect of insult [F(1,68) = 0.003, *p* = 0.96] or treatment [F(3,68) = 0.78, *p* = 0.51]. We did not find an interaction between insult and treatment [F(3,68) = 1.21, *p* = 0.32] (Figure 7D). We did not find a main effect of insult [F(1,68) = 0.93, *p* = 0.34] or treatment [F(3,68) = 1.18, *p* = 0.32] on the IL-6 expression levels. We did not find an interaction between insult and treatment [F(3,68) = 1.93, *p* = 0.13] (Figure 7E). There was no main effect of insult [F(1,68) = 1.25, *p* = 0.27] or treatment [F(3,68) = 0.32, *p* = 0.81] on the TNFα expression. We did not find an interaction between insult and treatment [F(3,68) = 0.47, *p* = 0.70] (Figure 7F).

### 3.8. Western Blotting Results

**Figure 8:**
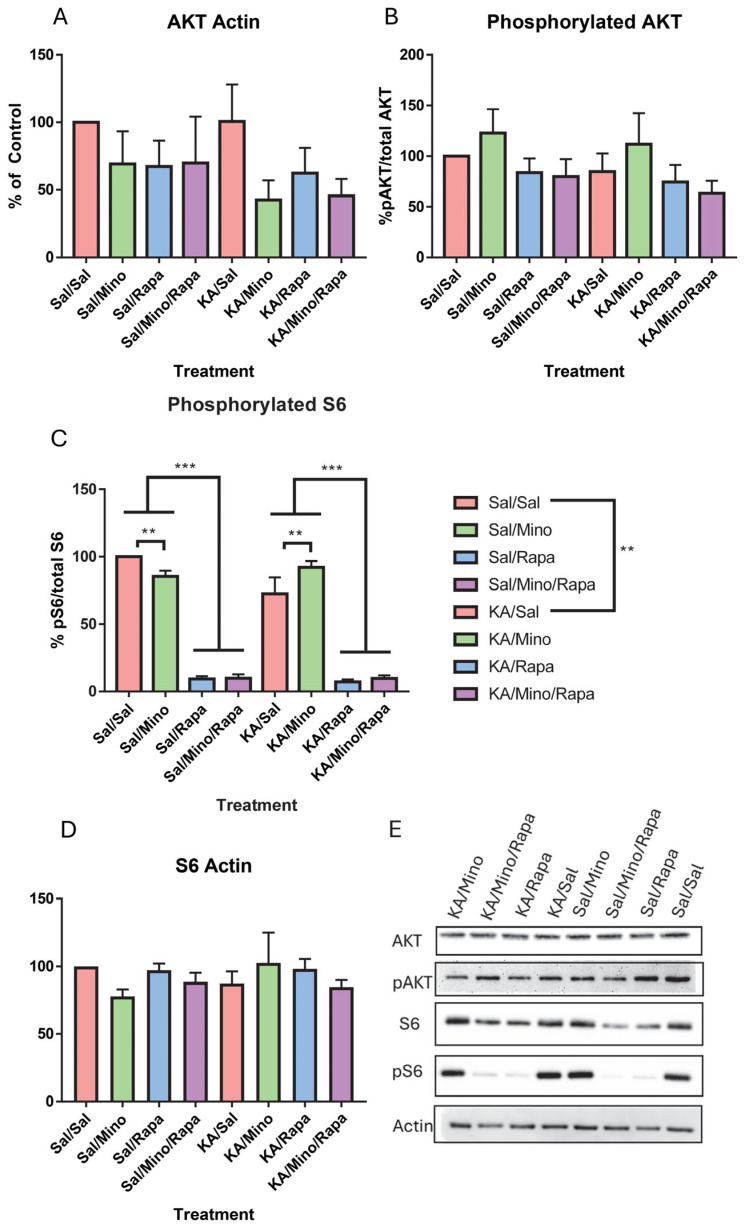
Rapamycin decreased mTOR signaling. Protein levels of total AKT (**A**) ratio of pAKT to AKT and (**B**) protein levels. The ratio of pS6 protein levels to total S6 levels (**C**). Protein levels of S6 to actin (**D**). Representative Western blots for AKT, tAKT, S6, pS6, and actin (**E**). Data are presented as the mean ± SEM. ** *p* < 0.01; *** *p* < 0.001. Saline (Sal), kainic acid (KA), minocycline (M), and rapamycin (R).

To investigate changes in the mTOR pathway, Western blotting was run on hippocampal tissue to analyze the amount of protein present. A two-way ANOVA on the relative amount of AKT to actin showed no effect of insult [F(1,56) = 0.85, *p* = 0.36] or treatment [F(3,56) = 1.92, *p* = 0.14]. There was no interaction found between insult and treatment [F(3,56) = 0.21, *p* = 0.89] (Figure 8A). We did not find an effect of insult [F(1,56) = 0.98, *p* = 0.33] or treatment [F(3,56) = 2.38, *p* = 0.08] on the amount of pAKT relative to AKT. We did not find an interaction between insult and treatment [F(3,56) = 0.02, *p* = 0.99] (Figure 8B). We did not find a main effect of insult [F(1,56) = 2.57, *p* = 0.12] on the amount of pS6 relative to S6. We did find a main effect of treatment [F(3,56) = 159.96, *p* < 0.001]. The mice treated with minocycline had higher pS6 than those treated with both minocycline and rapamycin and those treated with only rapamycin. The mice treated with both minocycline and rapamycin had less pS6 than those treated with saline. The mice treated with rapamycin had less pS6 than those treated with saline. We also found an interaction between insult and treatment [F(3,56) = 4.36, *p* = 0.01]. Pairwise comparisons showed that there was a significant difference in pS6 levels in control mice and those induced with KA and then treated with saline. In both the KA-induced group and the control group, there was a difference in pS6 levels between the mice treated with minocycline and saline (Figure 8C). We did not find a main effect of insult [F(1,56) = 0.12, *p* = 0.74] or treatment [F(3,56) = 0.46, *p* = 0.71] on the protein levels of total S6 compared to actin. We did not find an interaction between insult and treatment [F(3,56) = 1.34, *p* = 0.27] (Figure 8D). The Western Blot images can be found in Supplementary Material File S1.

## 4. Discussion

Neonatal seizures are associated with poor prognosis and adverse neurological outcomes [4]. Status epilepticus in infants, even without developmental delay, can interfere with normal psychomotor development, and about 16% of these children develop learning disabilities [38]. Seizures can increase susceptibility to later developing epilepsy and other comorbidities, including ASD [2,6]. The mTOR pathway has arisen as a potential mechanism underlying this association. In our study, we aimed to investigate whether there is a causal link between SE and ASD-like behavior, and the effectiveness of rapamycin and/or minocycline in rescuing these behaviors in female mice. We found that seizures resulted in social deficits and increased anxiety-like behaviors. We did not find any differences in locomotor activity, exploratory behavior, repetitive behaviors, or fear learning. While we did not find any changes in inflammation markers in the cortex or hippocampus, we did find altered mTOR signaling in the hippocampus. Rapamycin, as expected, significantly decreased the mTOR signaling, and seizure induction modulated the mTOR signaling.

For this study, we exclusively focused on females to improve the translational validity, uncover sex-dependent neural mechanisms, and address known clinical gaps. For many years, female mice were believed to show more variability than males, but recent research does not support this assumption [39,40]. However, women report more adverse side effects of antiseizure medications and are at an increased risk of developing treatment-resistant epilepsy [41,42]. Female mice show several differences from males regarding immune function. Females have lower IL-6 expression compared to males when given the lipopolysaccharide, which induces inflammation [43]. Males have more microglia than females on PD 4 in the hippocampus, amygdala, and parietal cortex, which then flips during adulthood [44]. Females have been shown to have increased basal mTOR phosphorylation compared to males [45]. Estrogen and progesterone modulate neuroinflammatory signaling, reduce cytokine expression, and influence microglial activation [21,22]. With all of these differences in mind, we focused the experiments on female mice.

One of the core features of autism spectrum disorder is social deficits. Mice are naturally social animals and can accurately discriminate between social and nonsocial stimuli [46]. One of the most commonly used paradigms to assess social function, the three-chamber social test, can reflect willingness to engage socially and social recognition abilities [47]. We found that SE significantly decreased sociability in female C57BL/6J mice. The female mice induced with KA spent less time interacting with the novel mouse and explored the chambers less than the control mice. Treatment with rapamycin or minocycline did not rescue this behavior. Other models of genetic and acquired epilepsy have shown significant social deficits [18,48,49,50]. Our laboratory has previously found reduced social interactions and socialization deficits from flurothyl- and KA-induced seizures [31,34]. A tuberous sclerosis model in rats, and inducing seizures with KA on PD 7 and 14, showed reduced social exploration behavior and increased social avoidance, similar to what we found [51]. However, all of these studies examined multiple seizures at different age ranges and/or were done exclusively in male rodents. Our study demonstrates that social deficits can arise after only one instance of SE in the neonatal stage in female wildtype mice.

The role of rapamycin and/or minocycline in reversing social deficits is unclear. We did not find an effect of rapamycin or minocycline on social behavior in our study. Other researchers have found mixed results on rapamycin’s effectiveness [52,53]. We have previously found that continued rapamycin treatment can increase sociability in neuron subset-specific PTEN knock-out mice (NS-PTEN KO) [54]. NS-PTEN KO mice are a common mouse model that presents with spontaneous recurrent seizures and autistic-like behaviors. Talos and colleagues [18] also showed that pretreatment with rapamycin 24 h before and 1 h after exposure to hypoxia was able to ameliorate autistic-like social behavior deficits in male rats. These studies all looked at the effects of repeated pretreatment with rapamycin. It could be that one instance of rapamycin treatment was ineffective in alleviating these deficits, or that pretreatment with rapamycin is more effective than posttreatment with the drug. The effectiveness of minocycline in reducing social deficits is widely unknown. In human uncontrolled clinical trials of FXS patients, minocycline has shown promise in improving language, attention, and social communication [55]. In mouse models of FXS, chronic minocycline treatment was shown to improve social recognition memory [56]. The inconsistency in our results could be because of differences in mouse strains or in the dosage and length of treatment. Our data posits that one posttreatment injection of minocycline and rapamycin is insufficient in female mice. Subsequent research could investigate different dosages and lengths of treatment that are required.

One of the most common comorbidities for patients with ASD or epilepsy is an anxiety disorder. Up to 40 percent and 30 percent of individuals diagnosed with autism spectrum disorder or epilepsy, respectively, also have an anxiety disorder diagnosis [57,58]. In the current study, we found changes in anxiety-like behavior. Female mice induced with KA spent more time in the closed arms than control mice in the EPM task. The effect of SE on anxiety-like behavior is mixed. Some researchers have found changes in anxiety in rats with KA-induced SE on PD 7 [49,59], while others have shown increased anxiety in Wister rats induced with pilocarpine and TSC model rats induced with KA [50,51]. Our laboratory has also found conflicting results. Wildtype mice induced with flurothyl showed no anxiety-like behaviors, while those induced with KA displayed increased anxiety [31,60]. The conflicting data could arise from differences in behavioral paradigms or sex differences in behavior. Some studies have shown that female rodents exhibit less anxiety-like behaviors in elevated plus maze and open field tasks, while others have shown no significant sex differences in animal behavior [61,62,63]. Other differences could emerge from differences in animal strain and the seizure induction method. In the current study, we did not see any effects of rapamycin or minocycline on anxiety-like behavior. Previously, our laboratory demonstrated that repeated treatment of rapamycin to NS-PTEN KO mice does not impact anxiety-like behavior [54]. The current study suggests that one treatment of rapamycin is insufficient to decrease anxiety-like behaviors, or that rapamycin is more effective in models that already have dysregulated mTOR signaling.

We found no effects of SE on locomotion, exploratory behavior, or anxiety in the open field task. The consistent activity levels suggest that the behavioral differences we found were not due to motor impairments. Other models of early-life SE have found that seizures can cause a reduction in overall activity [50]. However, this study used pilocarpine on PD 9 in rats. Differences in the effect of SE on activity levels may reflect differences in the induction method and rodent model used. We did see some decreases in activity in the elevated plus maze task, specifically in the total distance traveled. Rapamycin decreased the distance traveled compared to our other treatment groups when the mice were induced with KA. Mice that went into SE and were treated with minocycline or both minocycline and rapamycin traveled further than those treated with just rapamycin. This is consistent with prior work we have done in the laboratory, showing that multiple rapamycin treatments significantly decreased exploratory behavior [54]. Repeated minocycline has been shown to increase exploratory behavior in the Theiler’s virus model of temporal lobe epilepsy [64]. Our study shows that one instance of treatment with minocycline and/or rapamycin is sufficient to produce these changes in behavior. Another interesting finding is that we found an increase in locomotor behavior in the EPM test, but not in the open field test. This may be due to an order of testing effect. A future study should test the mice in the EPM first, then the open field, to determine the effects of the order of testing on the anxiety and activity levels of the mice.

Another core feature of ASD is repetitive, restricted behaviors. Mice will perform some of these behaviors, including burying foreign objects, investigating small holes, and grooming themselves. Male rats induced with KA on PD 7 have been shown to have dysregulated repetitive behaviors. The rats had a reduced number of marbles buried, as they focused their attention on moving and burying a limited number of them [49]. As such, we expected to find similar deficits in our female mice in the marble burying and nose poke tasks. We did not find a change in repetitive behaviors. While we did see a significant increase in center nose pokes in the KA group compared to the control, none of our other measures showed a behavior change. Based on the EPM data, we expected a decrease in center hole pokes in the KA group. It may be that this measurement is more sensitive to locomotor activity. We have found conflicting results in our laboratory where male and female C57BL/6J mice that received 15 flurothyl-induced seizures during postnatal days 7–11 showed no changes in repetitive behaviors [30]. Alternatively, male FMR1 KO mice with KA-induced seizures on PD 10 showed significant increases in repetitive behaviors in the nose poke task [34]. Additionally, a repeated rapamycin treatment has been shown to correct repetitive behaviors in a PTEN mouse model [54]. A potential reason for these differences is the type of tasks used to measure the behaviors. One study proposed that repetitive behaviors can be categorized into two separate groups: lower-order motor actions and higher-order cognitive behaviors [65]. Lower-order motor actions, like stereotyped movements, are characterized by repetition of movement and are the most researched. Higher-order actions, like compulsions and rituals, are more complex and characterized by cognitive rigidity. Marble burying and nose poke are two tasks that assess lower-order motor actions. It has also been shown that there may be sex differences in the prevalence of restricted, repetitive behaviors. Female BTBR mice, a well-established mouse model of ASD, do not show behavioral inflexibility and lower-order restricted repetitive behaviors like male mice do [66]. Seizures may have a greater effect on higher-order repetitive behaviors, rather than lower-order ones. The treatments may not have shown an effect on these tasks because there were no deficits to correct.

We did not find differences in the freezing behavior in a novel context or in response to an auditory cue. It has been shown that SE can impair short-term memory and plasticity, hippocampal-dependent memory and plasticity, episodic and working memory, and spatial learning [31,59,67,68]. Rapamycin treatment can increase performance deficits from SE on Morris water maze and novel object recognition tasks in rats with pilocarpine-induced seizures [52]. These changes in memory are derived from alterations in glutamate receptors in the hippocampus, enhancing mGluR-mediated long-term depression [68,69]. The deficits do not seem to extend to amygdala-dependent contextual fear and recognition memory [59,60]. In the current study, we found that delayed fear conditioning resulted in the acquisition of contextual fear memories. It could be that other aspects of cognition were impacted that we did not measure. Rapamycin and/or minocycline may be able to show benefits in these cognition domains. We did not investigate the mGluR activity or expression in the hippocampus. Future studies could look at the impact of KA-induced SE on mGluR activity and its association with other measures of cognition, and the effectiveness of rapamycin or minocycline in mitigating these impairments.

Seizure activity induces brain inflammation, and increased inflammation can trigger further seizures [16]. In adult mice and rats, chemoconvulsants and electrical stimulation trigger a rapid induction of inflammatory mediators in the brain, lasting days to weeks after SE. Kainic acid-induced SE, specifically, causes the activation of hippocampal microglia for up to 7 days [19]. Minocycline can reduce initial seizure-induced microglial activation. We found no changes in IL-1β, IL-6, or TNF-α in the cortex or hippocampus one day post-seizure. Research has identified the role of IL-1β, TNF-α, and IL-6 in seizure generation [16]. Studies have reported increases in TNF-α and IL-1β up to 24 h post-seizures in the hippocampus [70,71,72,73]. Interleukin 6 was maximally increased six hours after KA-induced SE in rodents [74]. A single instance of treatment with minocycline may not be enough to sustain a depressed inflammatory response one day after seizures. We may not have seen an increase in inflammation because minocycline may have decreased cytokine levels right after treatment, and they returned to the baseline prior to tissue collection. We may have missed the peaks of the inflammatory response, about 6 h post-SE, and the immediate effect of minocycline or rapamycin [70,73]. Future studies could look at more acute and long-term time points, including right after SE, right after treatment, 6 h later, 24 h later, or 7 days post-seizure, to better investigate the effects of minocycline and SE on inflammation. In addition, we used the cortex tissue that overlies the hippocampus for our PCR experiments. Future studies could examine specific areas within the cortex to make more thorough analyses of neuroinflammation changes. In addition, future studies could examine changes in epigenetic processes and use video-EEG to verify whether the mice had alterations in epileptiform activity.

Immune function, specifically cytokine and microglia responses, is modulated by sex hormones. In mice exposed to LPS, females had lower IL-6 expression compared to males [43]. Decreased immune responses could be protective against autistic-like behaviors. Increased IL-1β, IL-6, and TNF-α are associated with stereotypical behaviors, and higher levels of these cytokines are found in ASD patients [75,76]. Males have more microglia than females at PD 4 in the hippocampus, amygdala, and parietal cortex, and this difference flips in adulthood [44]. The sex differences in microglial morphology and count in the brain during neonatal developmental periods could relate to specific susceptibility windows between males and females in neurodevelopmental disorders. Minocycline may be acting only on microglial activation, not completely blocking the inflammatory response [19]. Females having decreased microglia in development and decreased immune responses to insults may contribute to why we did not see an immune response to seizures or an effect of minocycline or rapamycin on our inflammatory markers.

The mTOR pathway is strongly activated after KA-induced SE [13]. The mTOR pathway is hyperactive within a few hours and up to 24 h after seizures in the hippocampus and neocortex [13,14,52]. There is a second peak of activation up to seven days later in the hippocampus [13,14]. Rapamycin treatment can block mTOR activation post-seizures and disrupt the development of epilepsy and autistic-like behaviors [13,18]. In our current study, we found that rapamycin inhibited mTORC1 activity and KA suppressed mTORC1 activity through a decrease in the pS6 levels. Females have been shown to have increased basal mTOR phosphorylation compared to males, and certain PI3K/AKT regulators and downstream targets, like PTEN, act in a sex-dependent manner [45]. The decrease in mTORC1 activity following SE could be from a ceiling effect in female mice. They already have increased mTOR signaling, so SE, instead of increasing further, caused a decrease in the signaling. This could be protective against SE-induced autistic-like behaviors. Increased mTOR activity in the brain is associated with ASD-related behavioral deficits, and differential AKT/mTOR signaling is seen in young children with ASD [17,77]. Despite seeing mTOR inhibition acutely, we did not see any effect of our treatments on our behaviors. One treatment of rapamycin and or minocycline was insufficient to normalize function in female C57BL/6J mice after one neonatal episode of SE. One consideration is that we used a sample size of five to six in our Western blotting experiments. This is a usual sample size in many Western blotting experiments, but it may not have been powerful enough to detect subtle effects [78]. While we observed reductions in pS6 that were consistent with rapamycin activity, we did not include a dedicated positive control group. A prior study demonstrates that rapamycin reliably reduces pS6 levels within hours of administration [13].

Other considerations in our work are that we only used a single dose at a single time point. There is a limitation in the use of a single-dose design. Future studies could include dose–response experiments to determine the optimal dose. The doses we used were based on prior studies. In addition, we targeted a time point where there is a known increase in cytokine activity and mTOR activation [13,16]. Both rapamycin and minocycline have time-dependent effects on signaling pathways and inflammation that may not persist long-term. In addition, it would have been beneficial to examine the long-term changes in mTOR signaling and levels of cytokines in adult animals. The acute effects may not mirror the long-term effects.

## 5. Conclusions

Our work demonstrates that a single episode of kainic acid-induced status epilepticus on postnatal day 10 results in long-term deficits in social and anxiety-like behavior. Our results show that status epilepticus can disrupt mTOR signaling and not immune responses in female mice. These signaling pathways, and rapamycin and minocycline, may interact differently in females than in males, highlighting the increasing need to include female subjects in research. The endogenous hormonal neuroprotection in females may have produced a “ceiling effect,” limiting the observable benefit of additional pharmacological intervention. Future research could investigate differences in behavior using different behavioral tasks. They could also explore other molecular markers like microglia activation, synaptic proteins, and mGluR to examine sex differences in the mechanisms behind epilepsy and autism spectrum disorder.

## Data Availability

All data generated or analyzed during this study are accessible through https://figshare.com, Cambridge, MA, USA.

## Supplementary Materials

The following supporting information can be downloaded at: https://www.mdpi.com/article/10.3390/neurosci7030055/s1, File S1: Western blots.

## Abbreviations

The following abbreviations are used in this manuscript:

ASD	Autism Spectrum Disorder
EPM	elevated plus maze
DFC	delay fear conditioning
FXS	Fragile X Syndrome
IL-1β	interleukin-1β
IL-6	interleukin-6
IL-8	interleukin-8
ITI1	Inter-trial interval 1
IP	intraperitoneal
KA	kainic acid
KO	knockout
mTOR	mammalian target of rapamycin
MB	marble burying
M	minocycline
NP	nose poke
OF	open field
PD	postnatal day
PTEN	phosphatase and tensin homolog
RT-qPCR	quantitative reverse transcription–polymerase chain reaction
R	rapamycin
SE	status epilepticus
TSC	Tuberous Sclerosis Complex
TNF-α	tumor necrosis factor-alpha
