# Supplementary figures and images for: Rapamycin and Minocycline Treatment Does Not Rescue Behavioral and Molecular Changes Induced by Early-Life Seizures in Female Mice
Source: NeuroSci. 2026 May 5;7(3):55. doi: 10.3390/neurosci7030055 (PMC13214891; doi:10.3390/neurosci7030055)

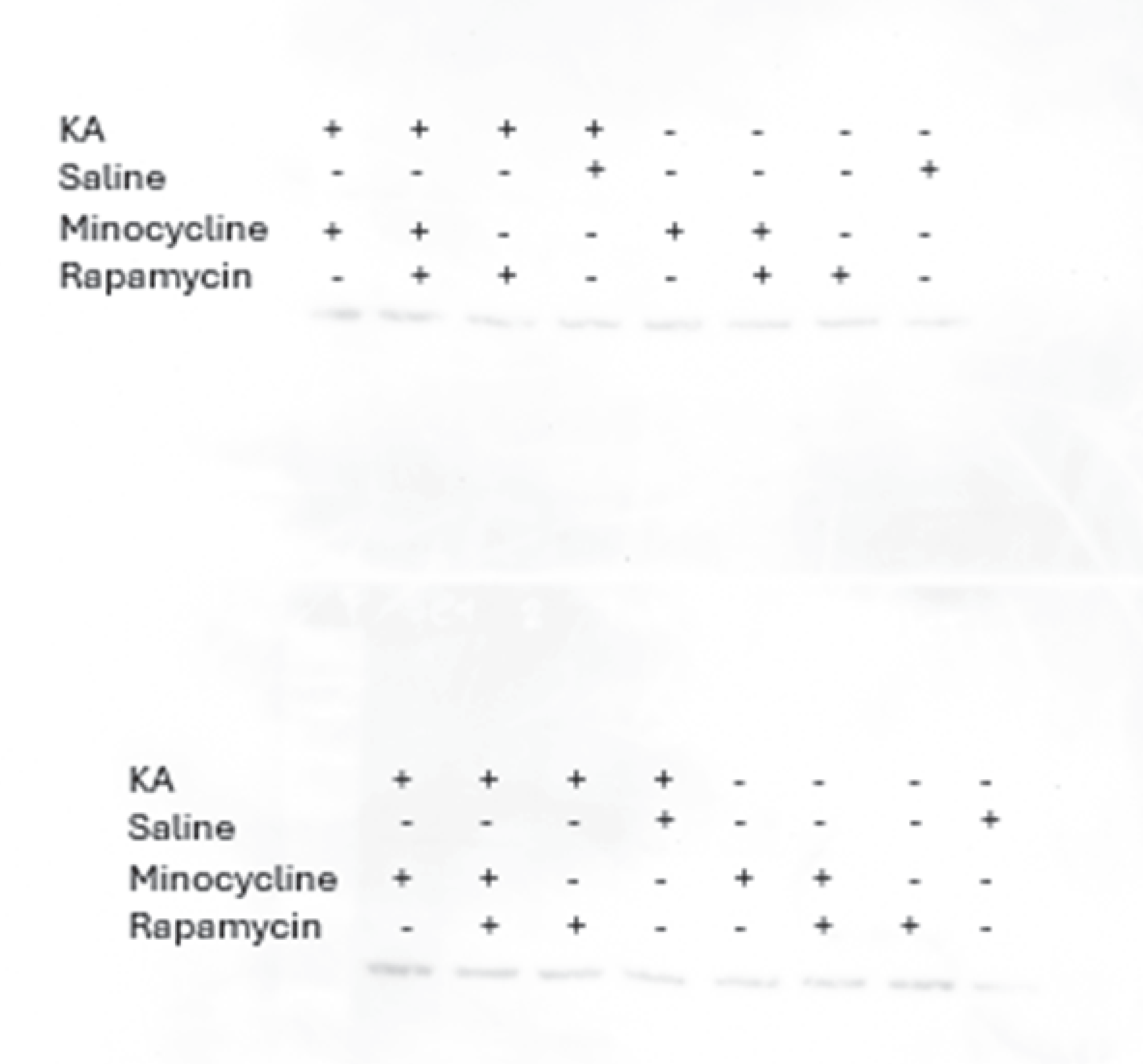

Supplement: Supplementary file 1 [file neurosci-07-00055-s001.zip › Western blots 4-7-26/1_2_AKTxactin_extrabri.tif]

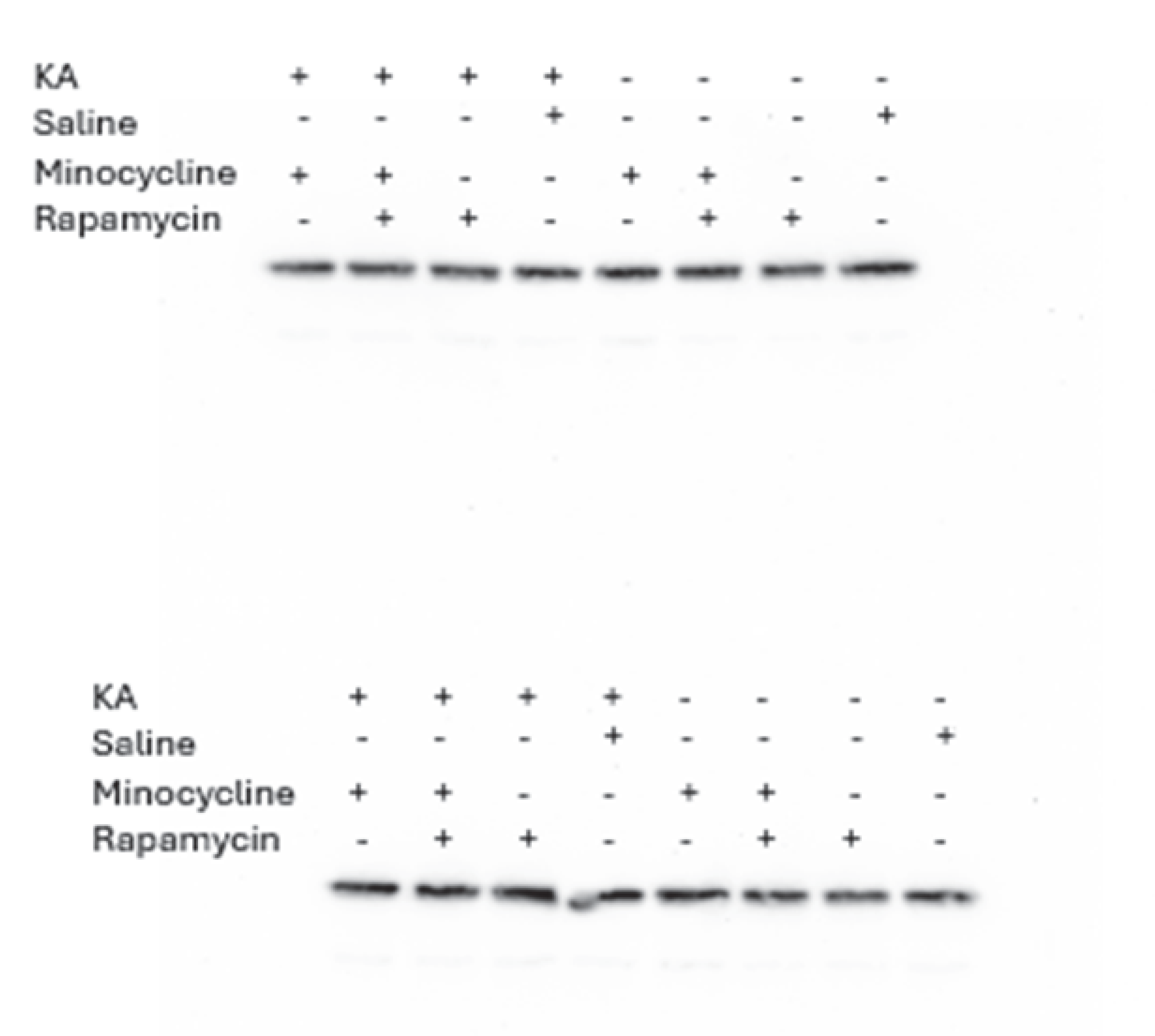

Supplement: Supplementary file 1 [file neurosci-07-00055-s001.zip › Western blots 4-7-26/1_2_AKT_bri.tif]

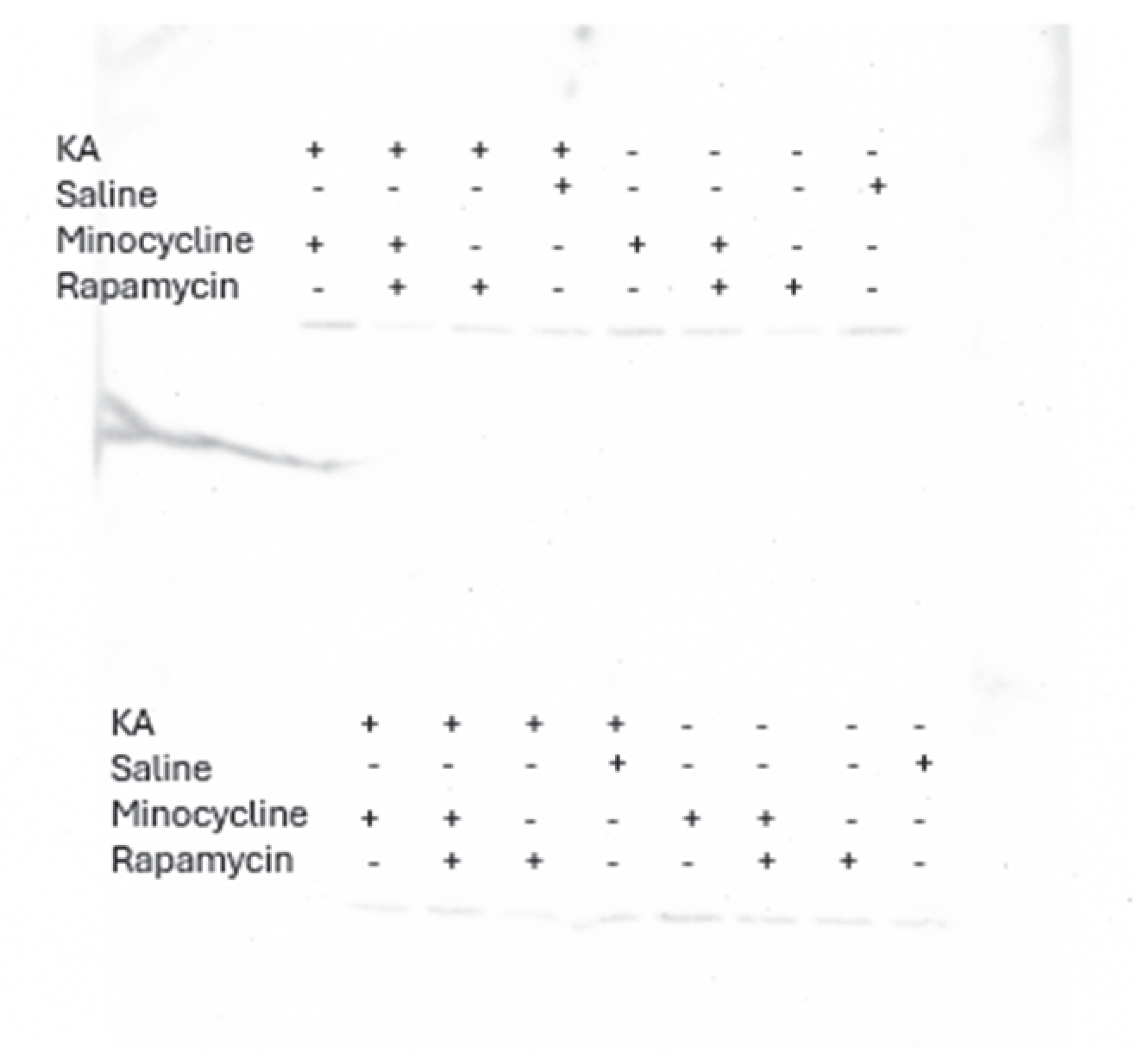

Supplement: Supplementary file 1 [file neurosci-07-00055-s001.zip › Western blots 4-7-26/1_2_pAKT_extrabri.tif]

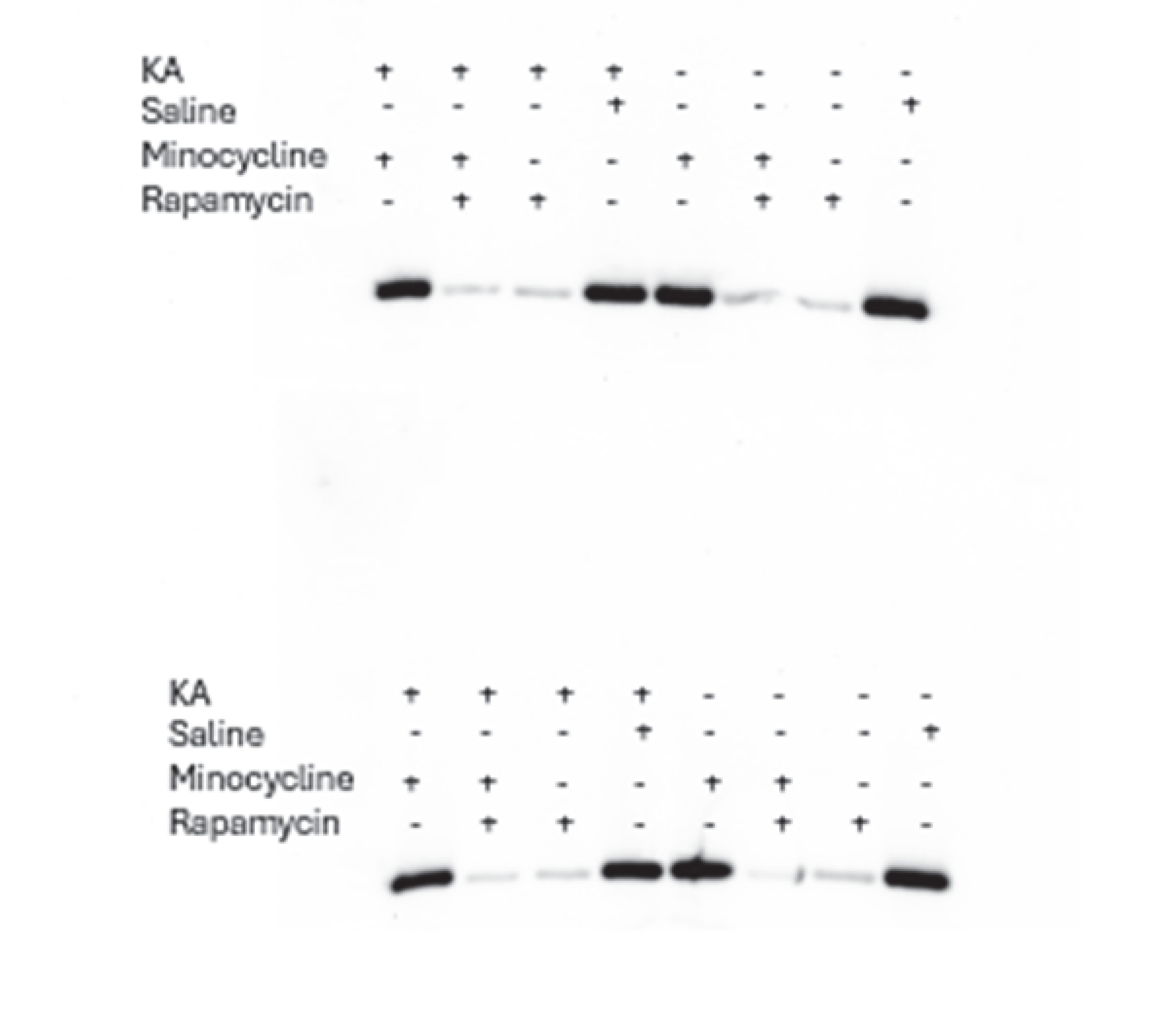

Supplement: Supplementary file 1 [file neurosci-07-00055-s001.zip › Western blots 4-7-26/1_2_pS6_med.tif]

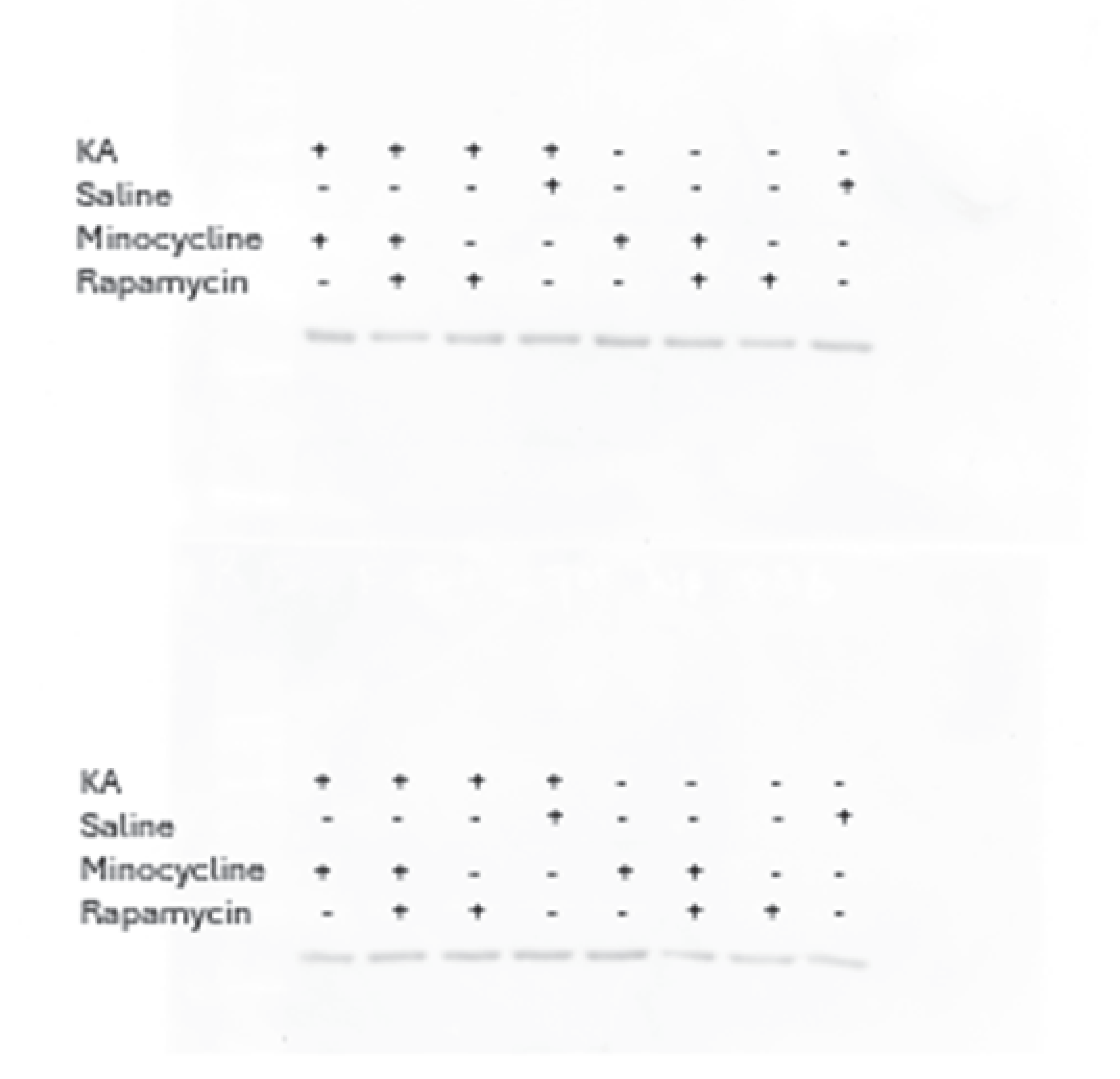

Supplement: Supplementary file 1 [file neurosci-07-00055-s001.zip › Western blots 4-7-26/1_2_S6xactin_light.tif]

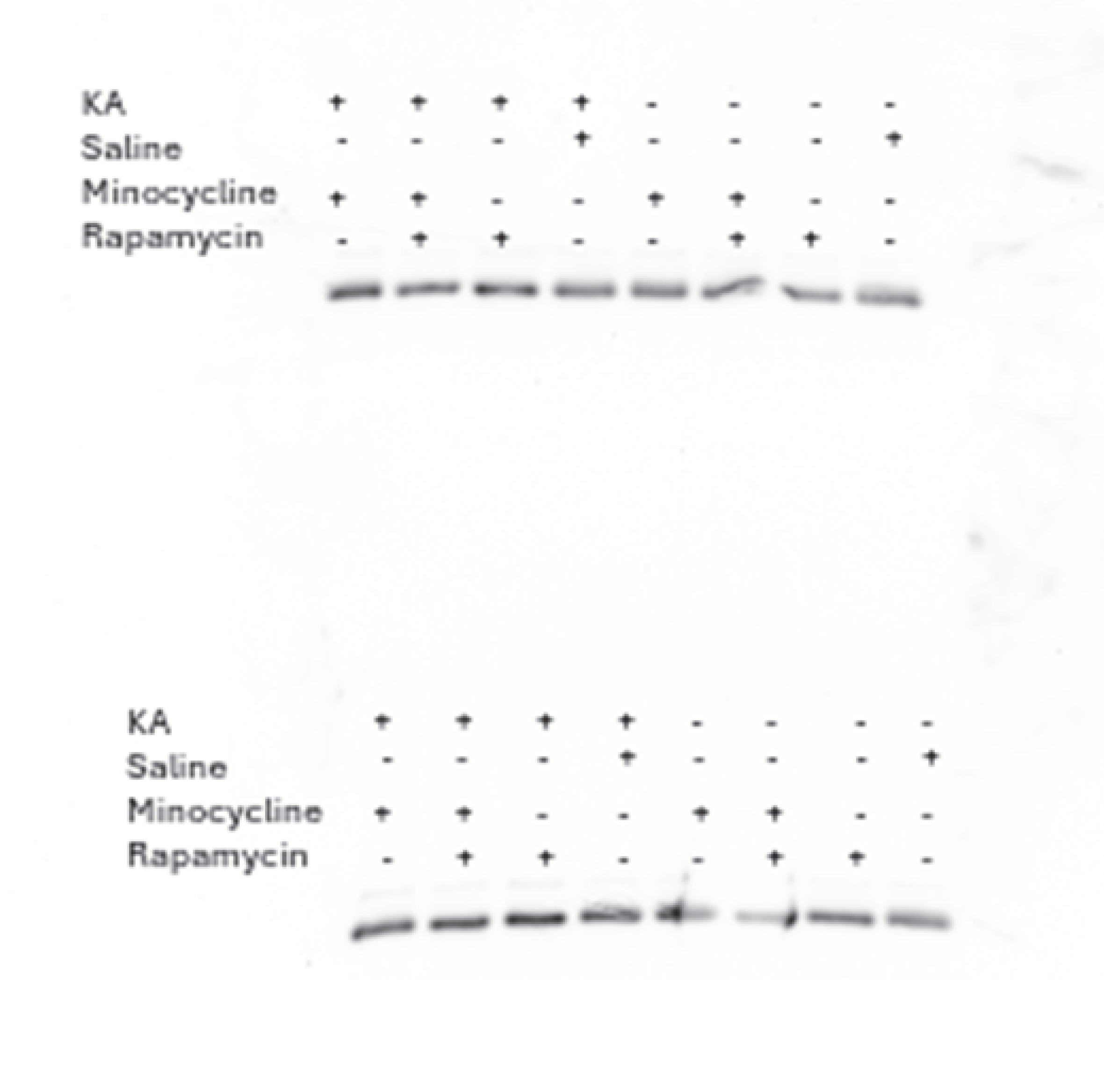

Supplement: Supplementary file 1 [file neurosci-07-00055-s001.zip › Western blots 4-7-26/1_2_S6_med.tif]

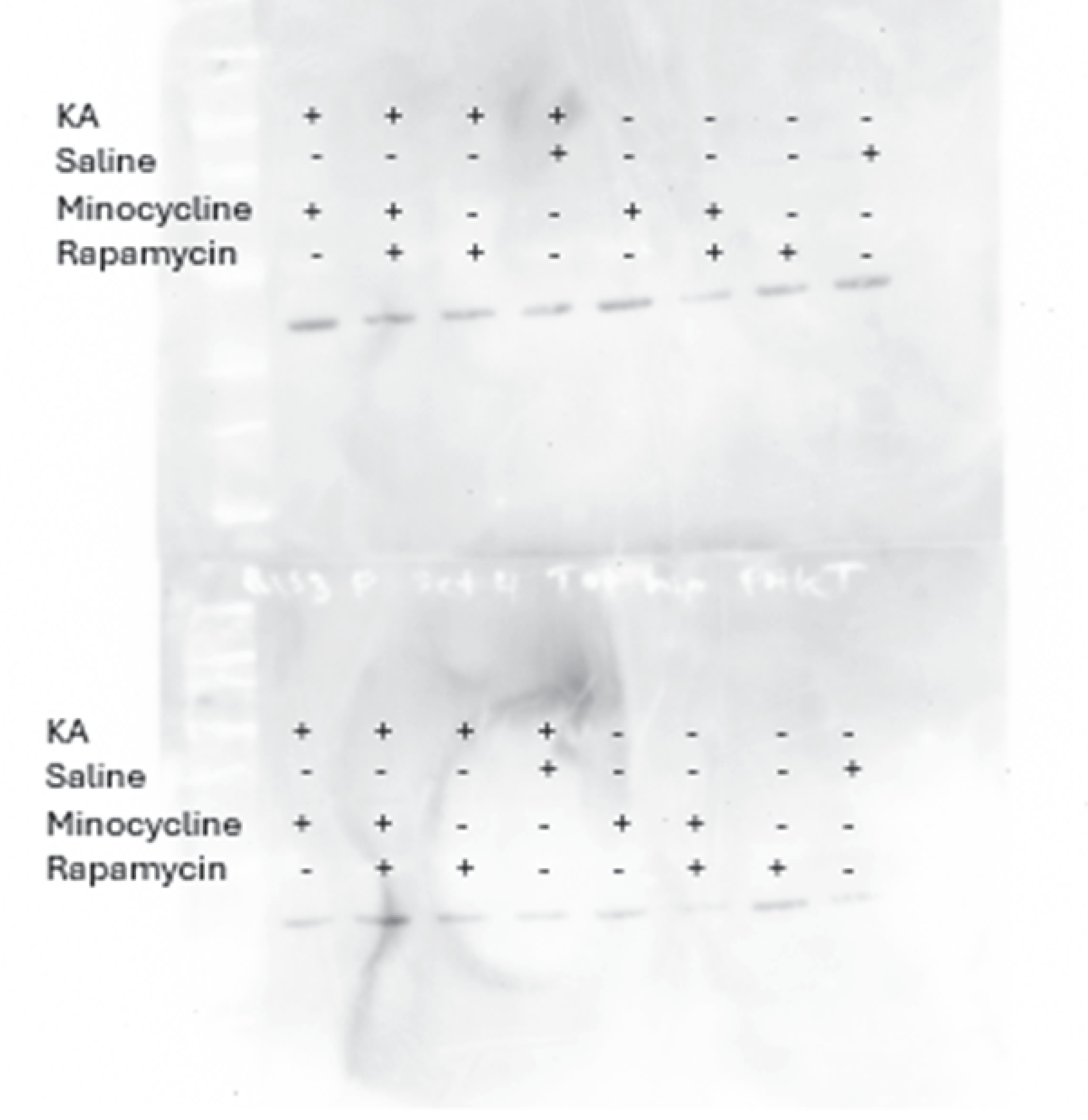

Supplement: Supplementary file 1 [file neurosci-07-00055-s001.zip › Western blots 4-7-26/3_4_AKTxActin_med.tif]

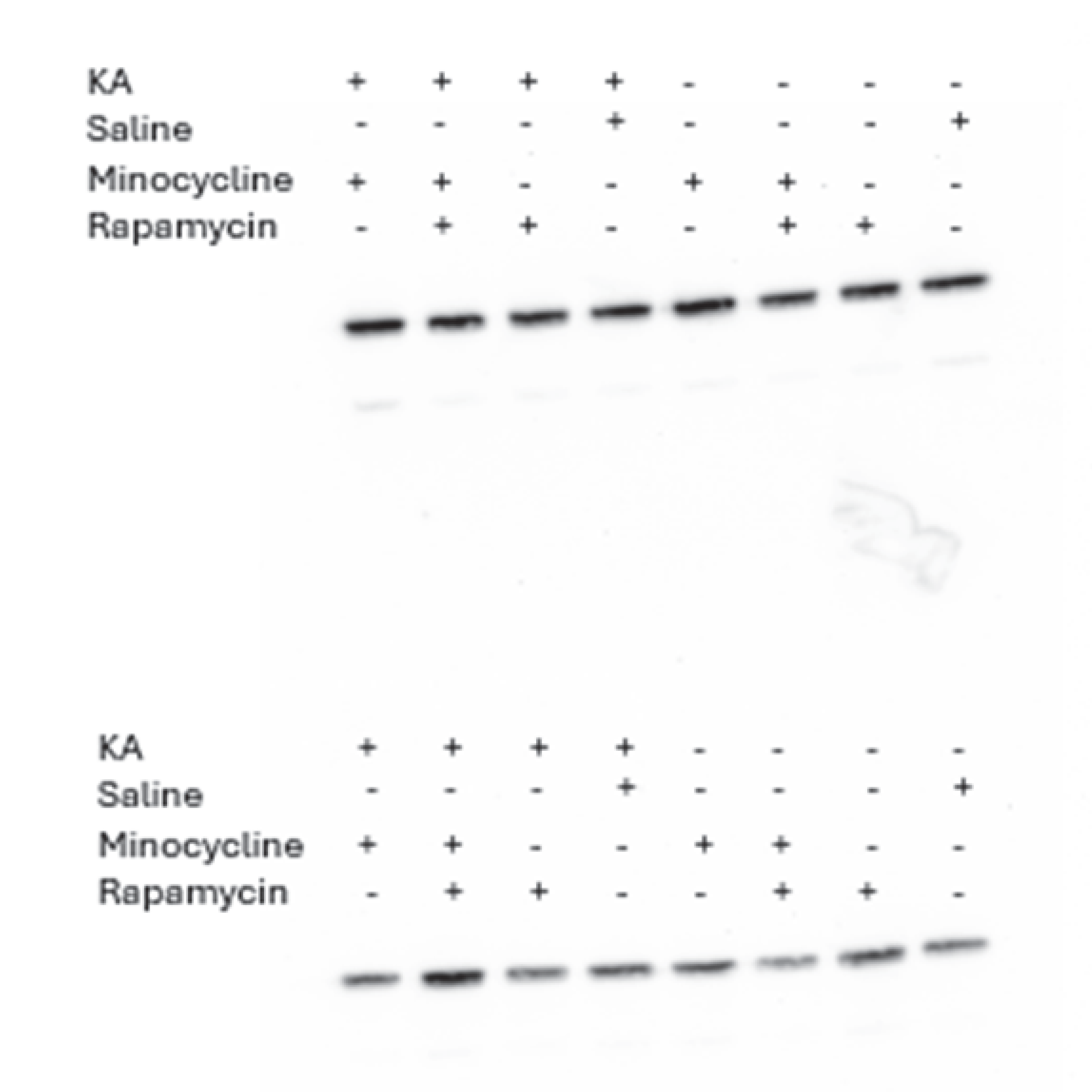

Supplement: Supplementary file 1 [file neurosci-07-00055-s001.zip › Western blots 4-7-26/3_4_AKT_bri.tif]

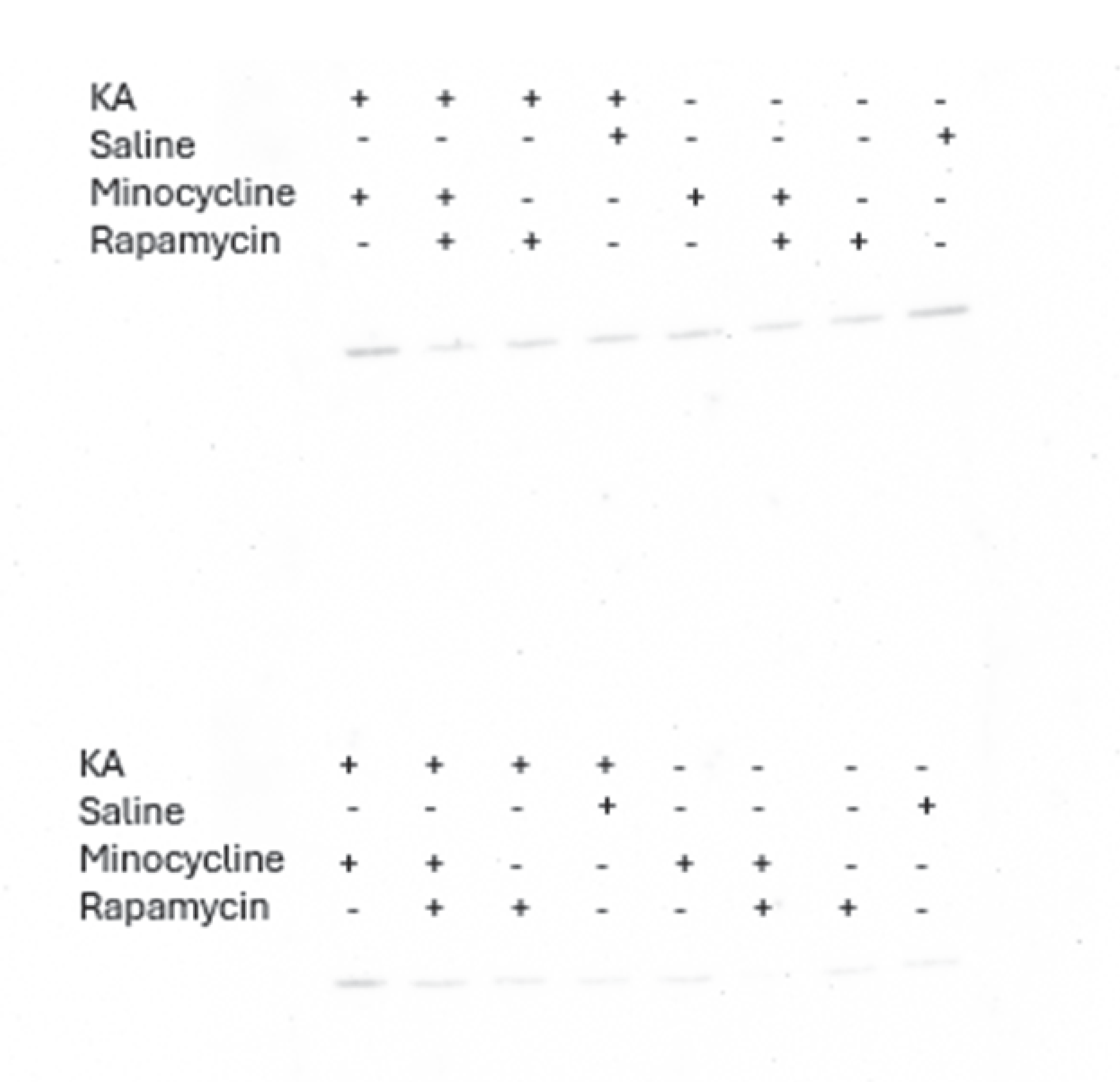

Supplement: Supplementary file 1 [file neurosci-07-00055-s001.zip › Western blots 4-7-26/3_4_pAKT_extrabri.tif]

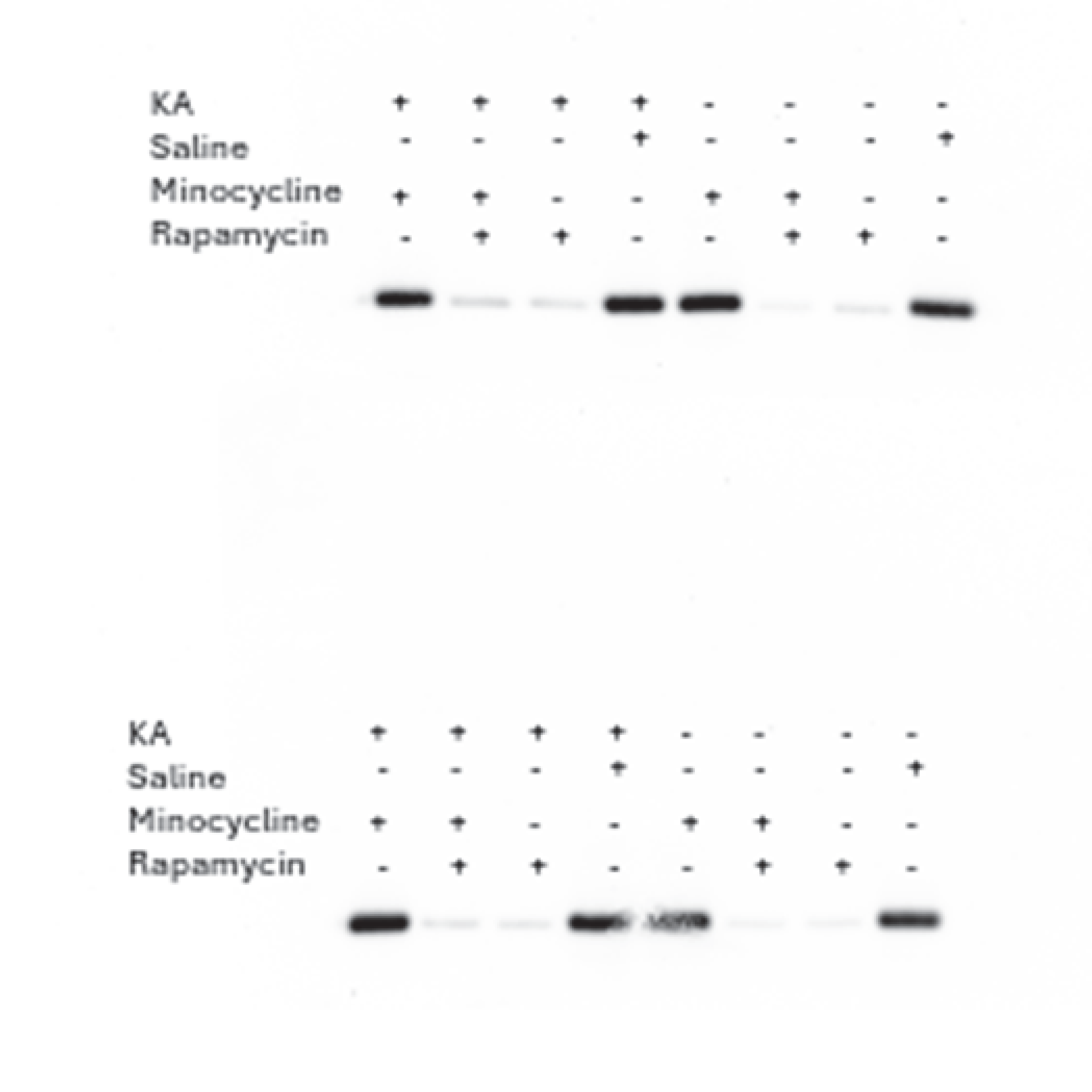

Supplement: Supplementary file 1 [file neurosci-07-00055-s001.zip › Western blots 4-7-26/3_4_pS6_med.tif]

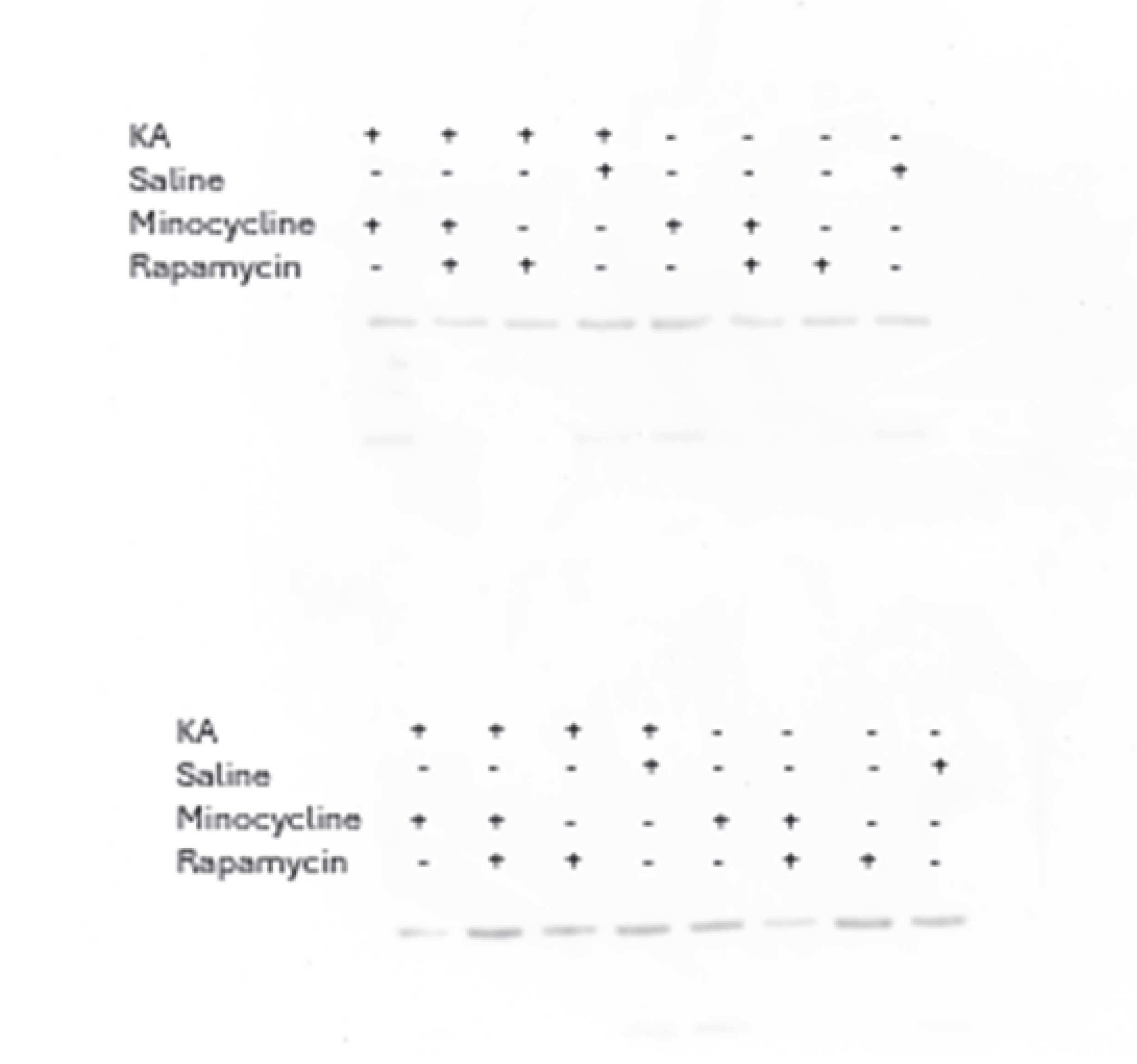

Supplement: Supplementary file 1 [file neurosci-07-00055-s001.zip › Western blots 4-7-26/3_4_S6xactin_med.tif]

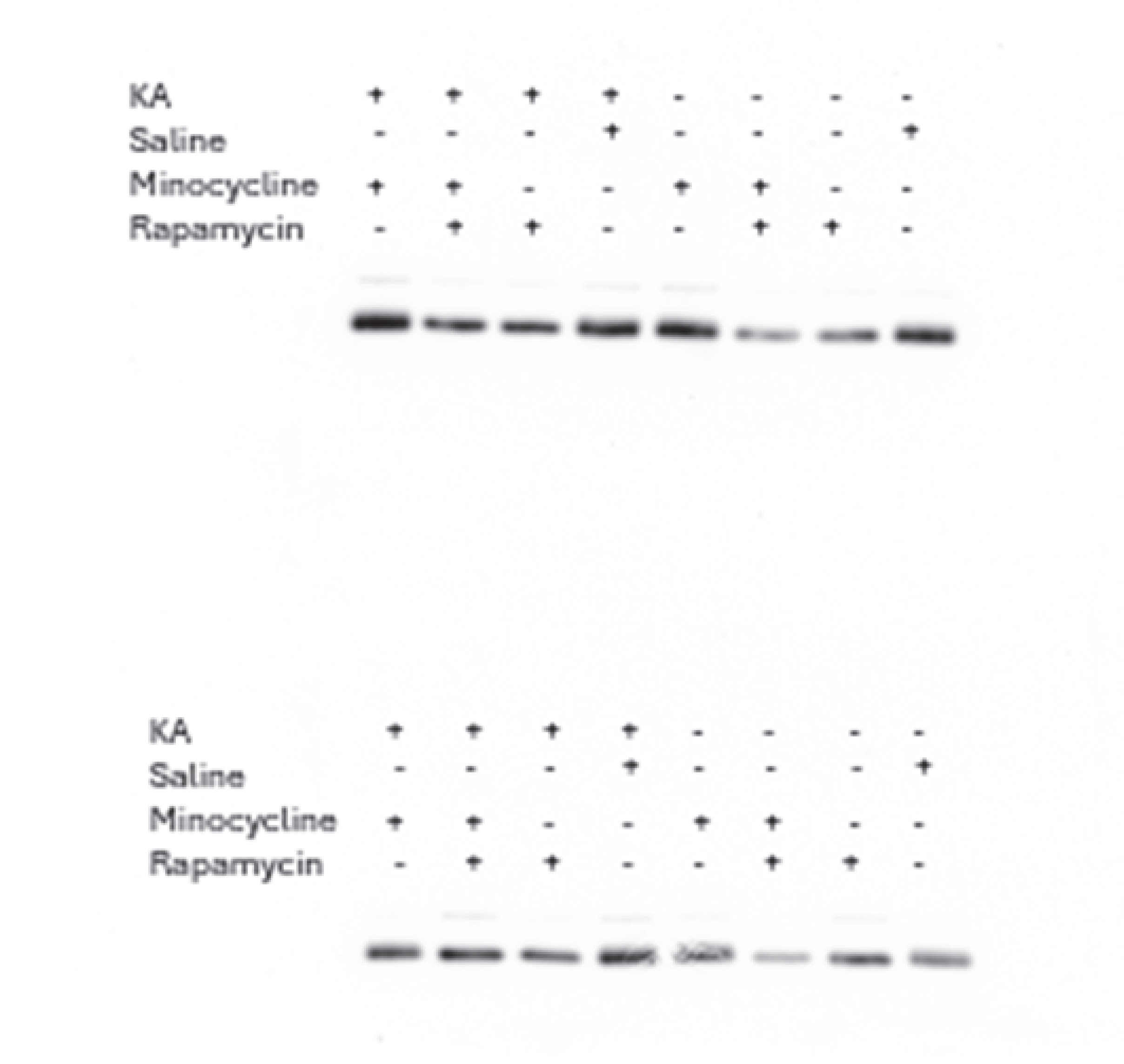

Supplement: Supplementary file 1 [file neurosci-07-00055-s001.zip › Western blots 4-7-26/3_4_S6_med.tif]

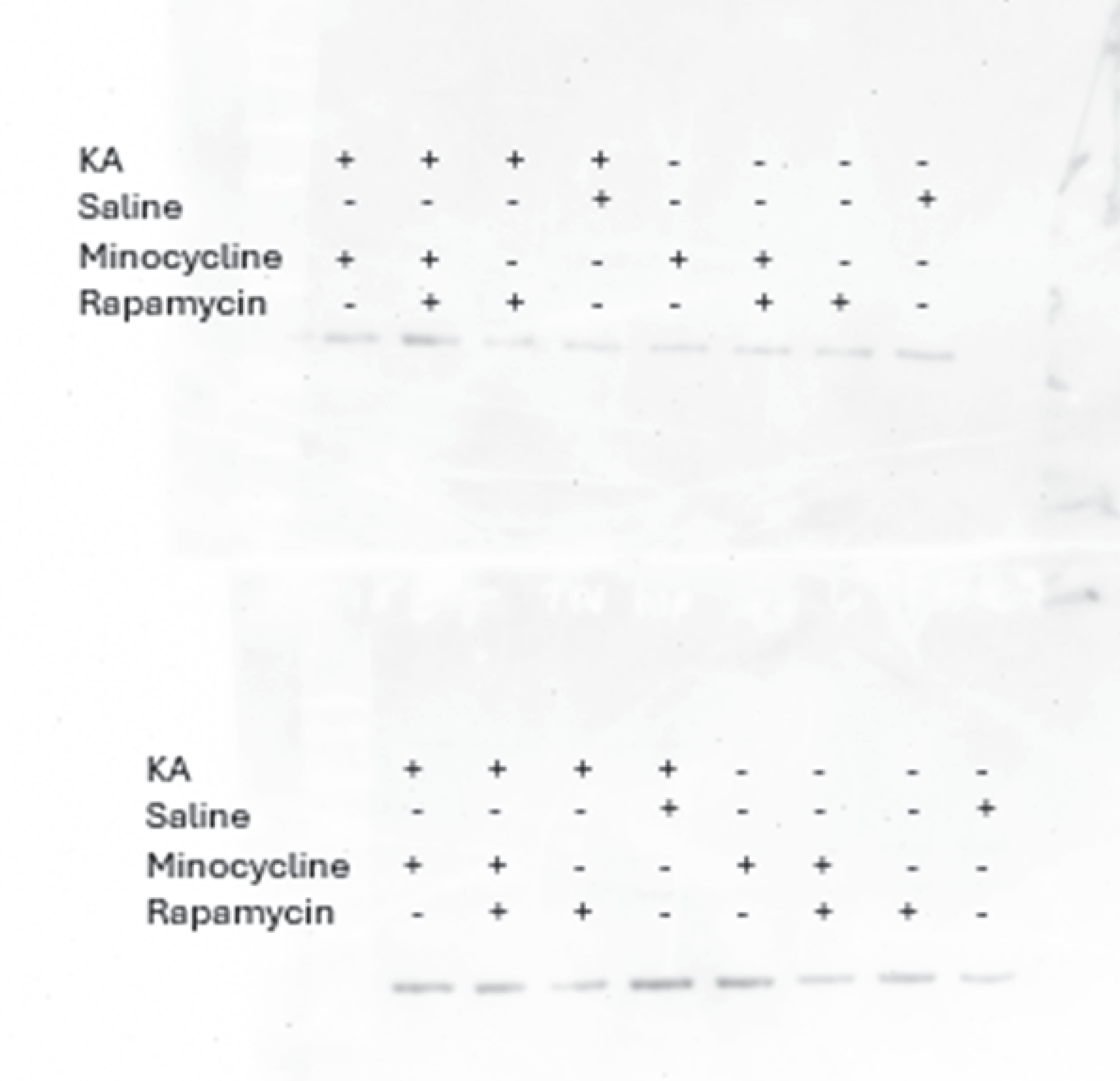

Supplement: Supplementary file 1 [file neurosci-07-00055-s001.zip › Western blots 4-7-26/5_6_AKTxActin_extrabri.tif]

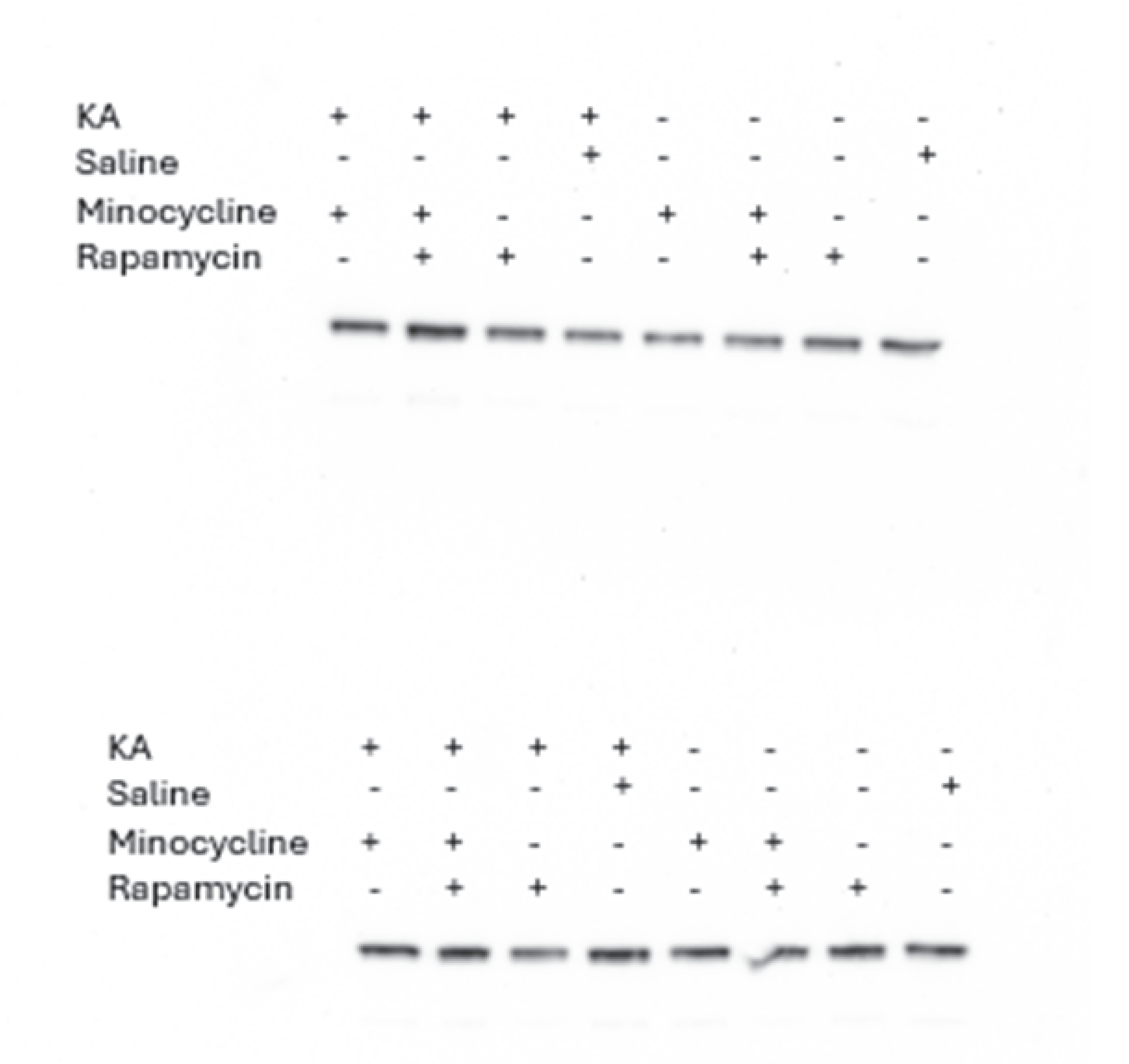

Supplement: Supplementary file 1 [file neurosci-07-00055-s001.zip › Western blots 4-7-26/5_6_AKT_bri.tif]

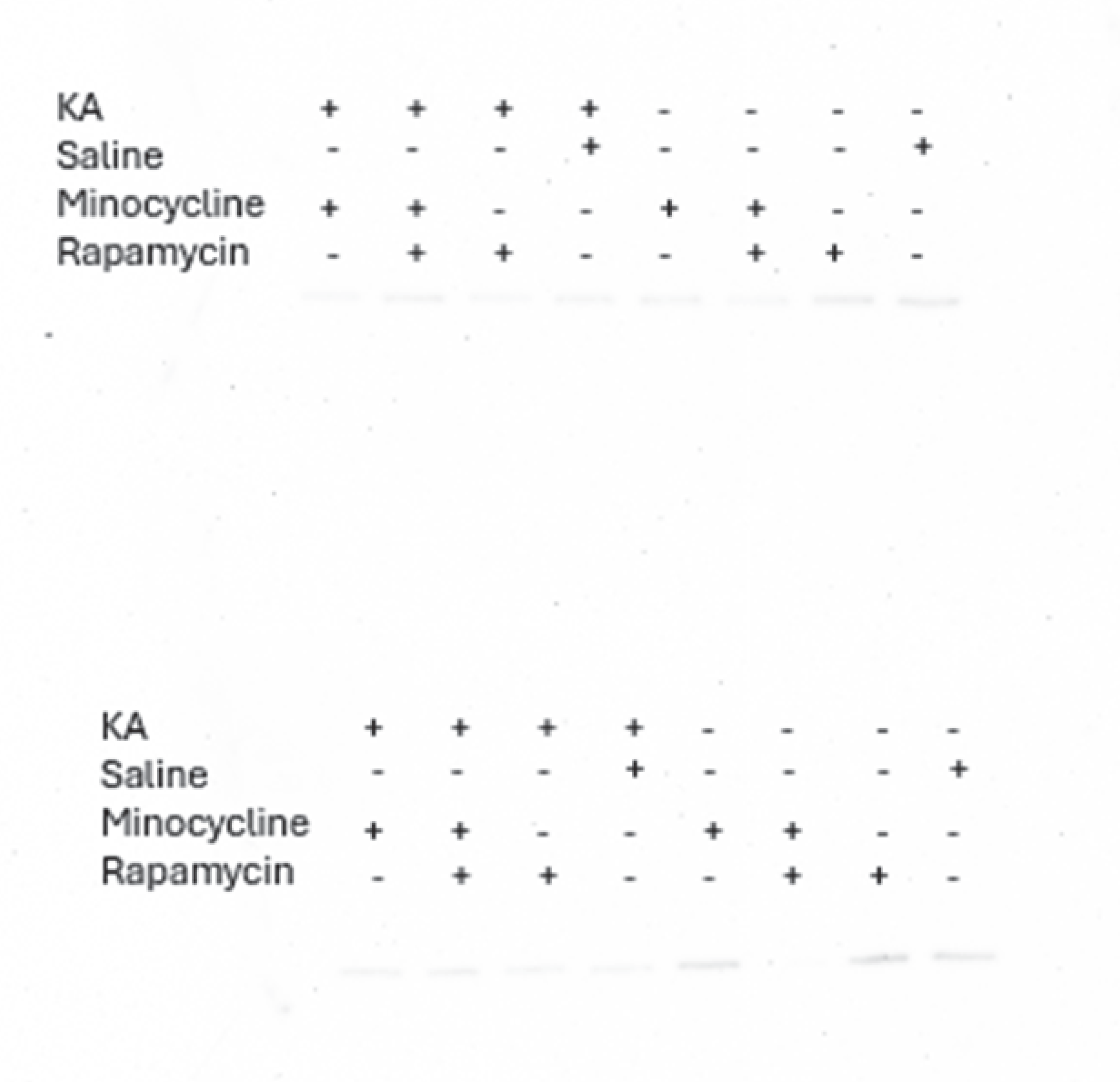

Supplement: Supplementary file 1 [file neurosci-07-00055-s001.zip › Western blots 4-7-26/5_6_pAKT_extrabri.tif]

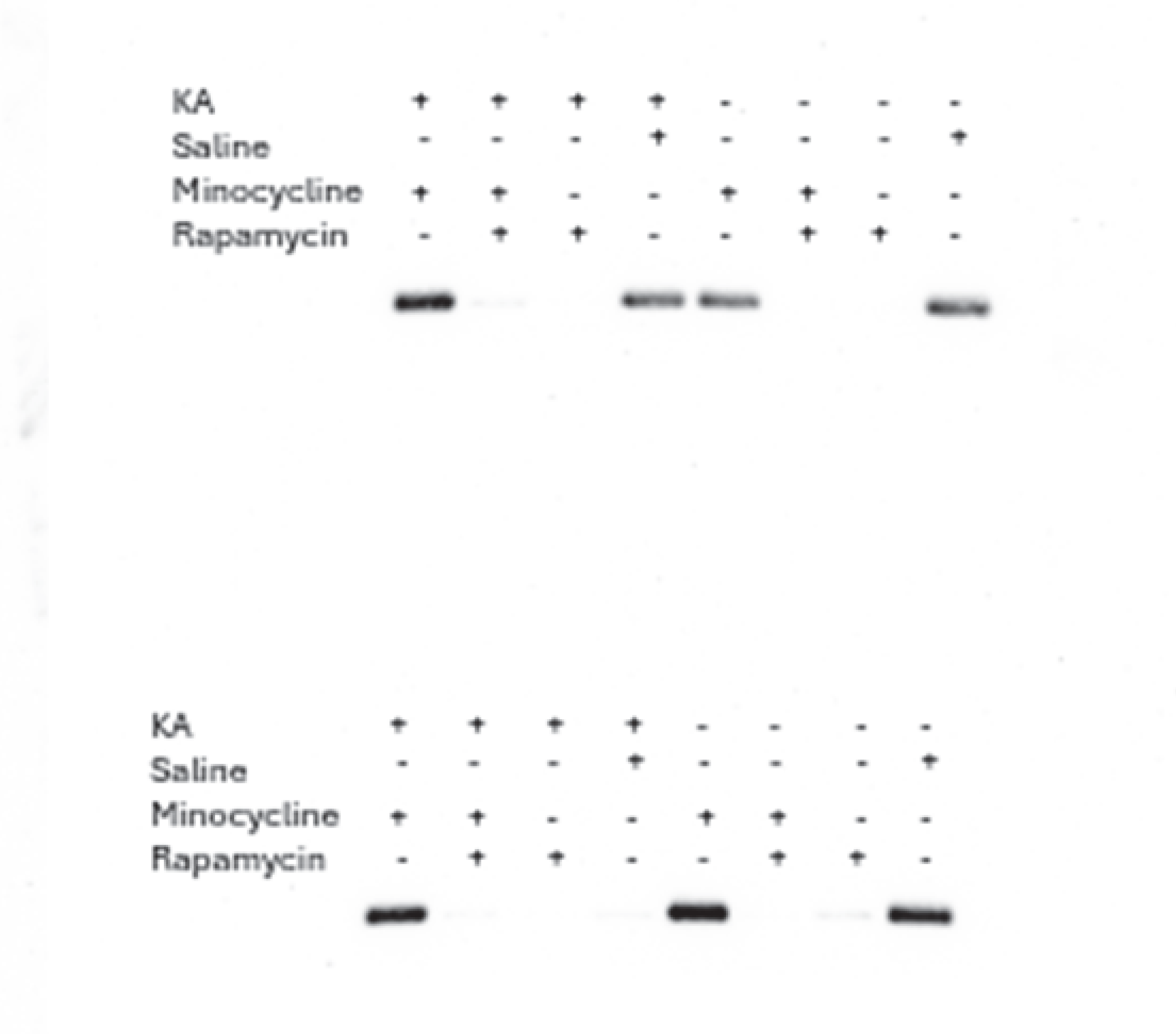

Supplement: Supplementary file 1 [file neurosci-07-00055-s001.zip › Western blots 4-7-26/5_6_pS6_bri.tif]

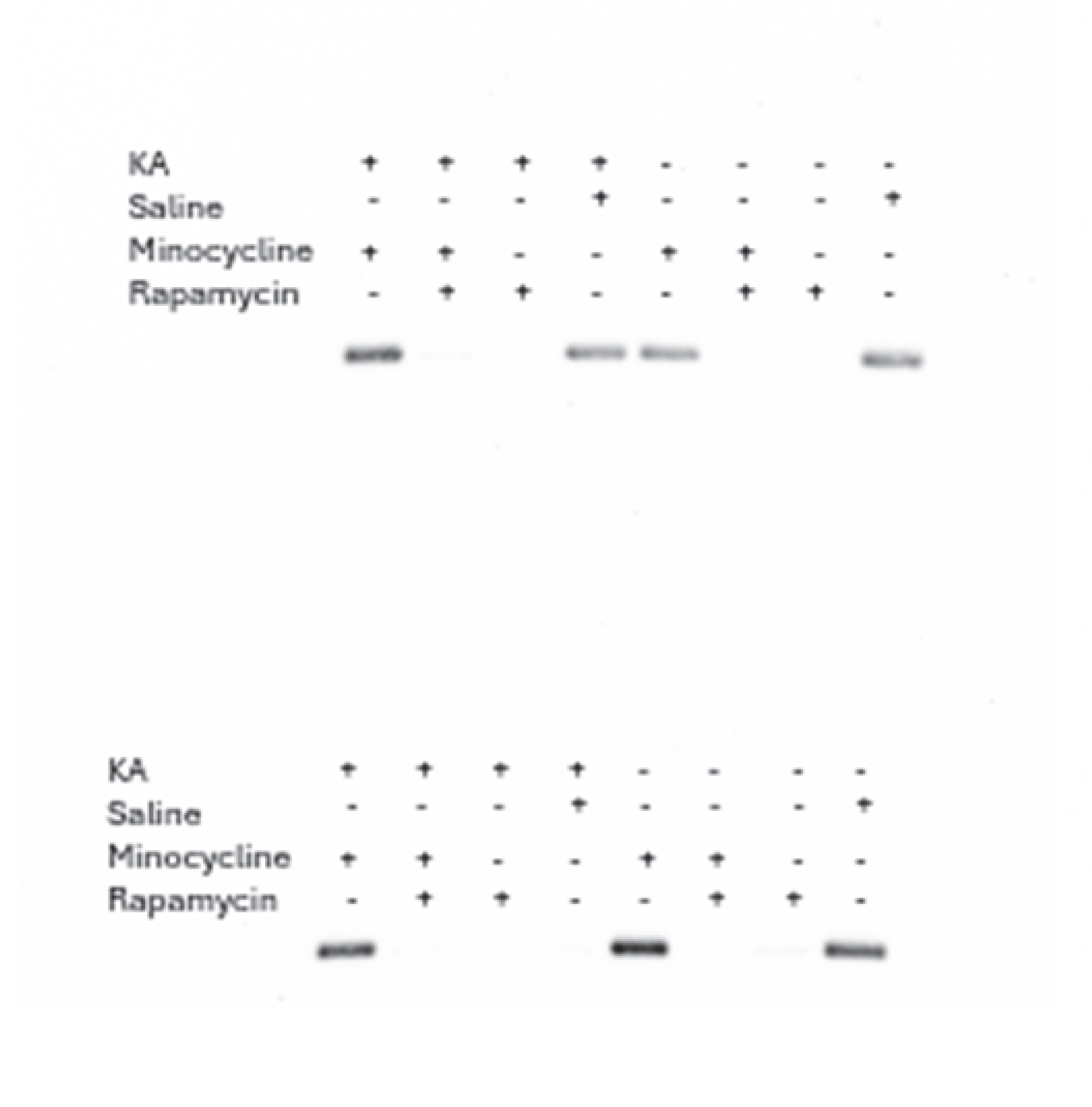

Supplement: Supplementary file 1 [file neurosci-07-00055-s001.zip › Western blots 4-7-26/5_6_ps6_med.tif]

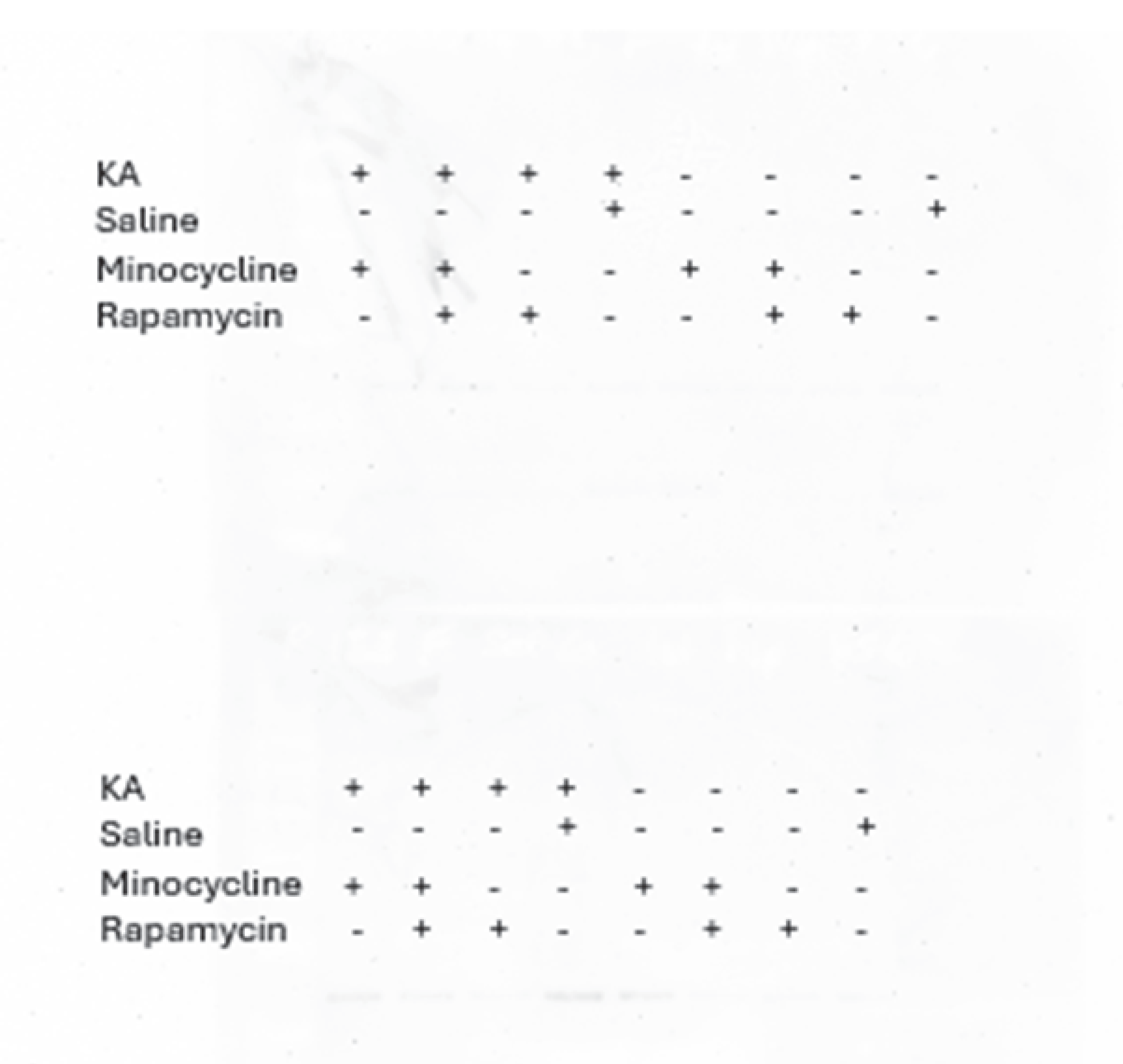

Supplement: Supplementary file 1 [file neurosci-07-00055-s001.zip › Western blots 4-7-26/5_6_S6xactin_extrabri.tif]

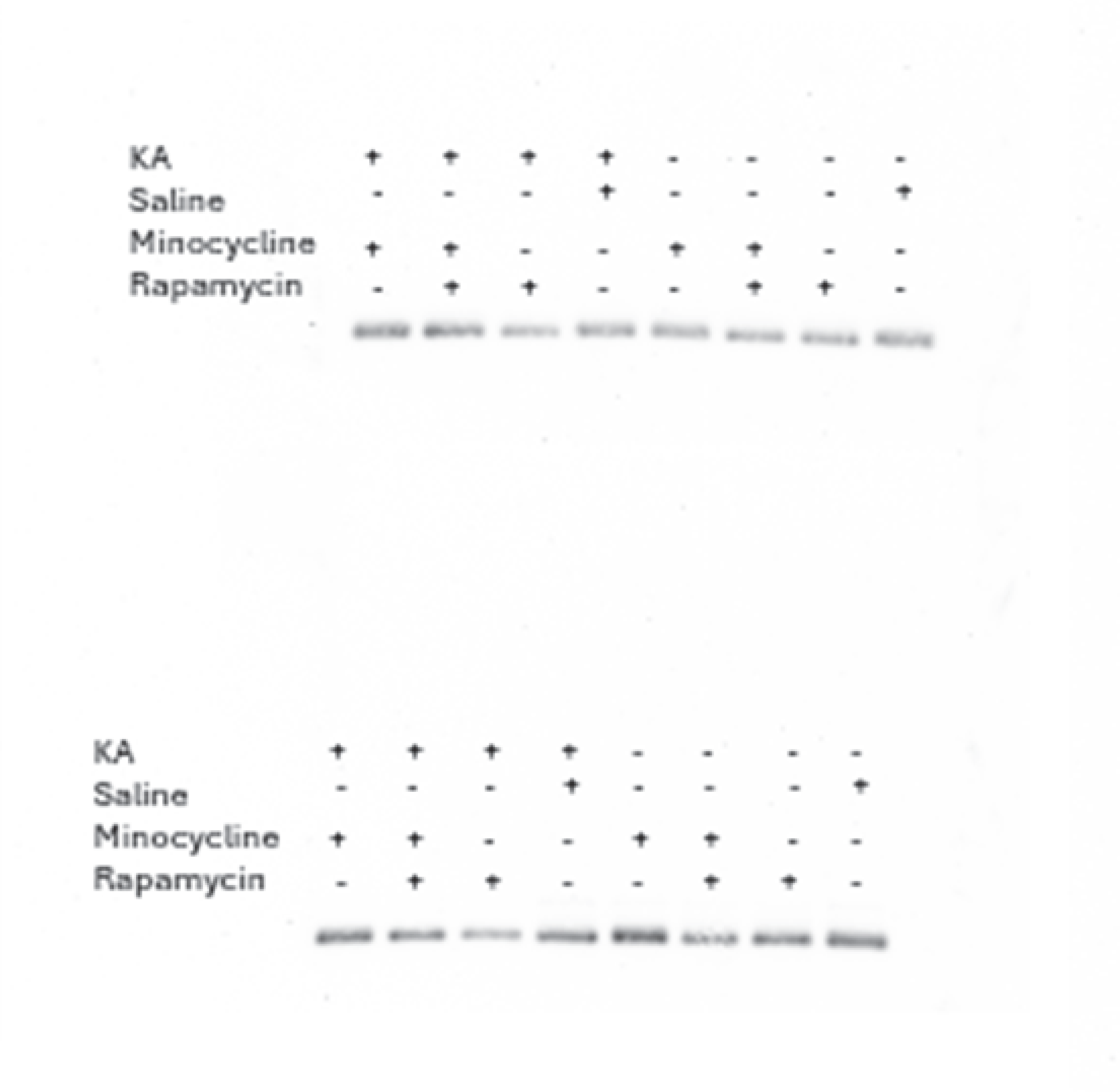

Supplement: Supplementary file 1 [file neurosci-07-00055-s001.zip › Western blots 4-7-26/5_6_S6_extrabri.tif]

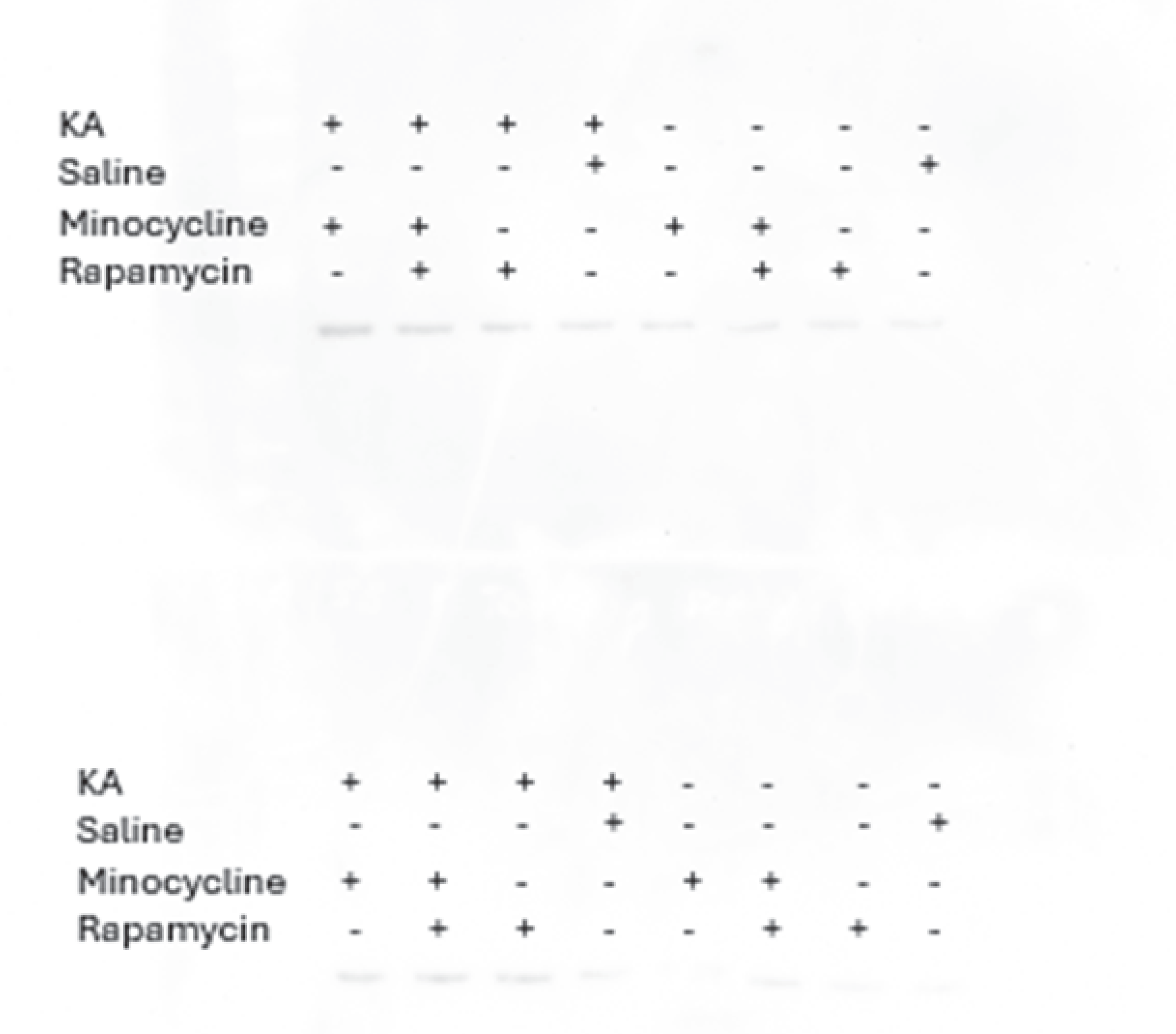

Supplement: Supplementary file 1 [file neurosci-07-00055-s001.zip › Western blots 4-7-26/7_8_AKTxActin_light.tif]

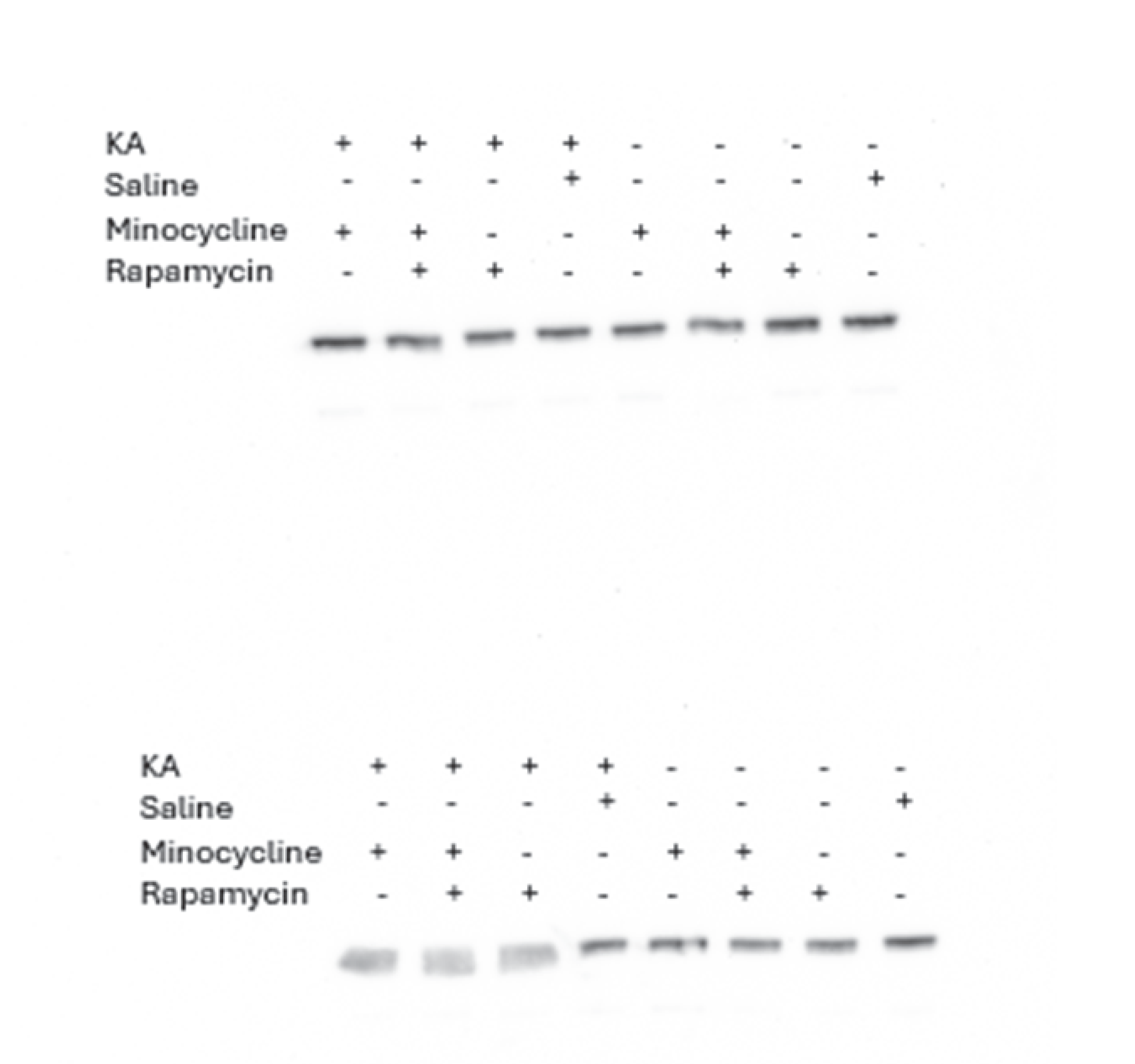

Supplement: Supplementary file 1 [file neurosci-07-00055-s001.zip › Western blots 4-7-26/7_8_AKT_med.tif]

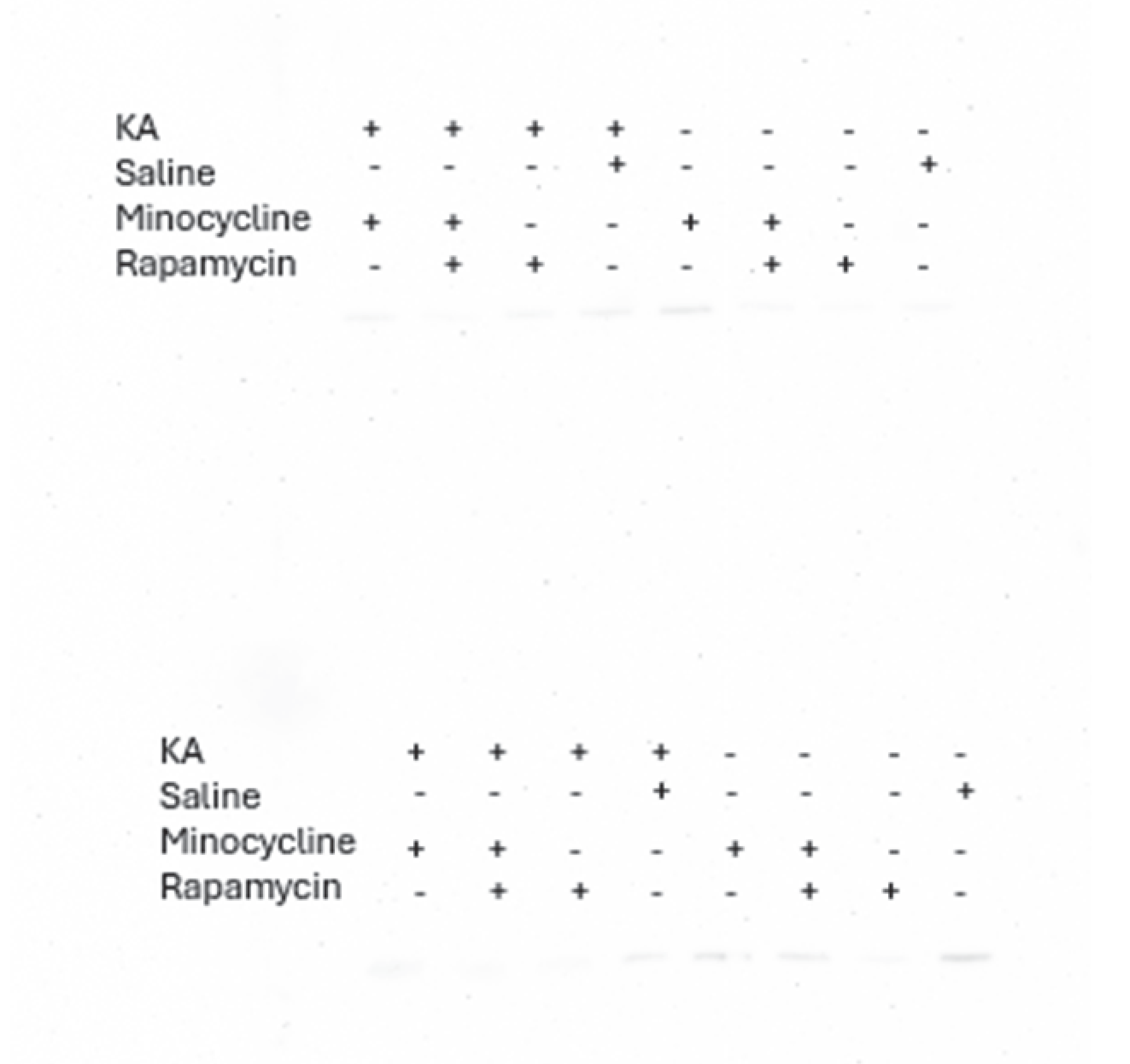

Supplement: Supplementary file 1 [file neurosci-07-00055-s001.zip › Western blots 4-7-26/7_8_pAKT_extrabri.tif]

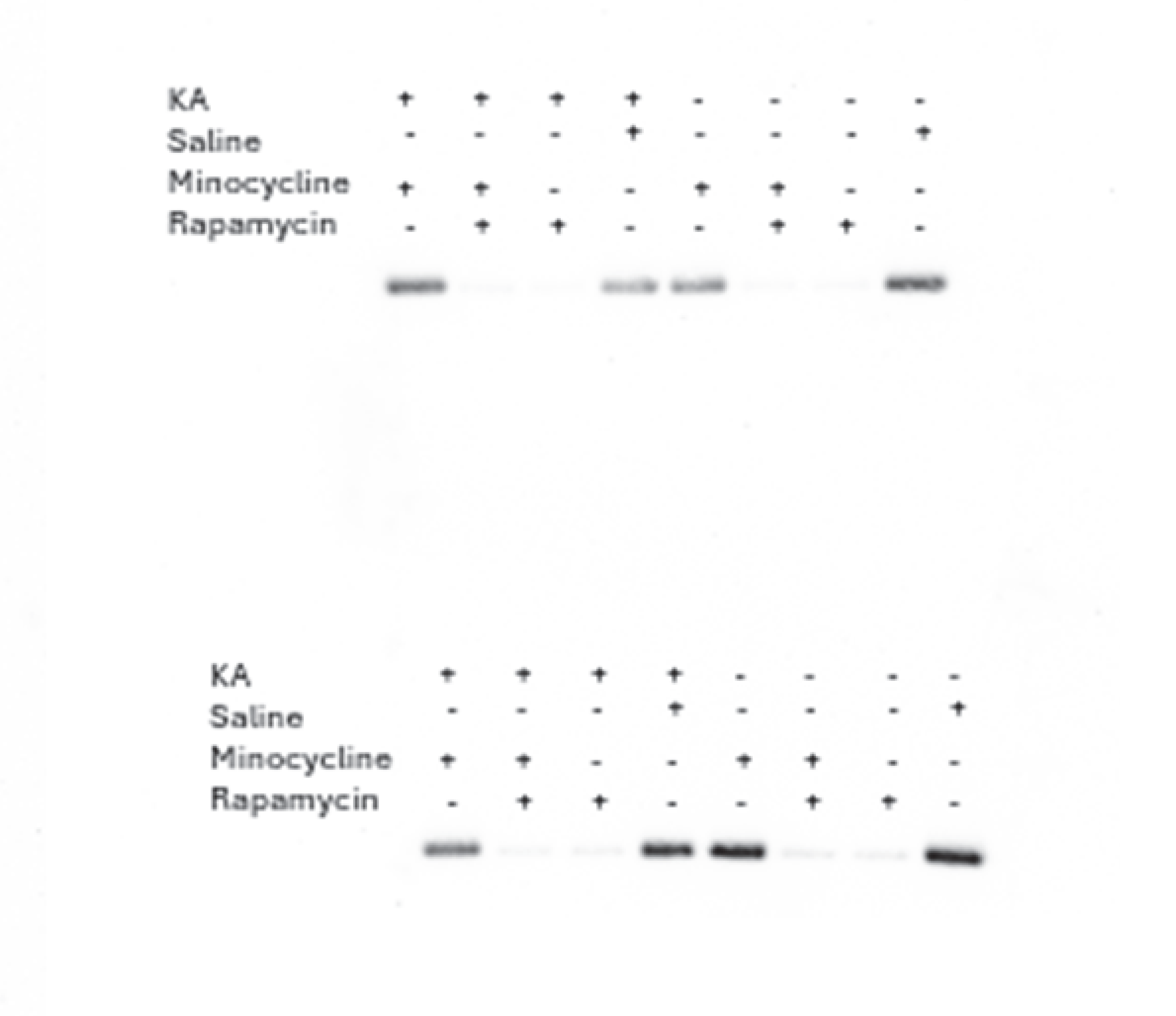

Supplement: Supplementary file 1 [file neurosci-07-00055-s001.zip › Western blots 4-7-26/7_8_pS6_med.tif]

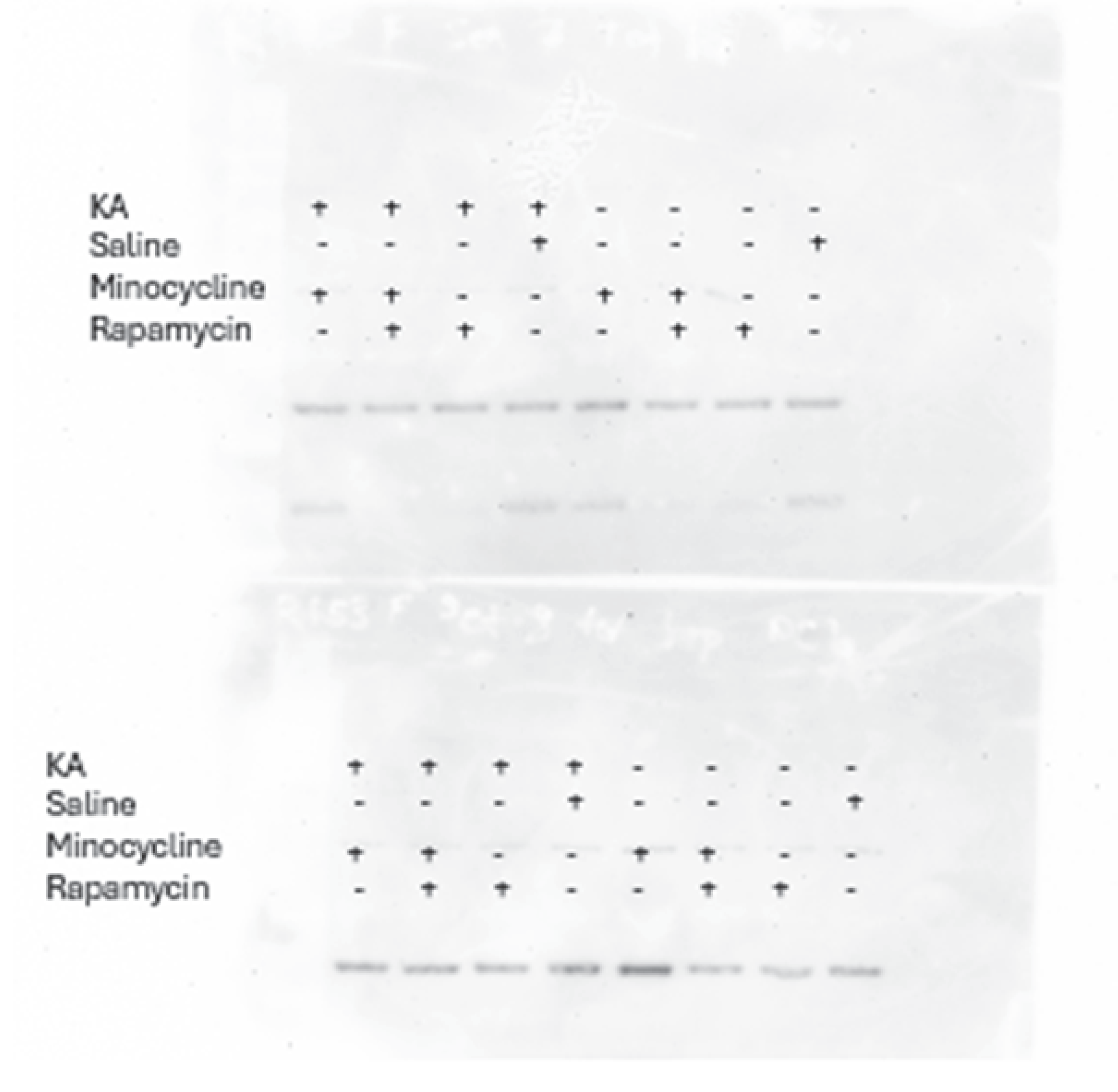

Supplement: Supplementary file 1 [file neurosci-07-00055-s001.zip › Western blots 4-7-26/7_8_S6xactin_extrabri.tif]

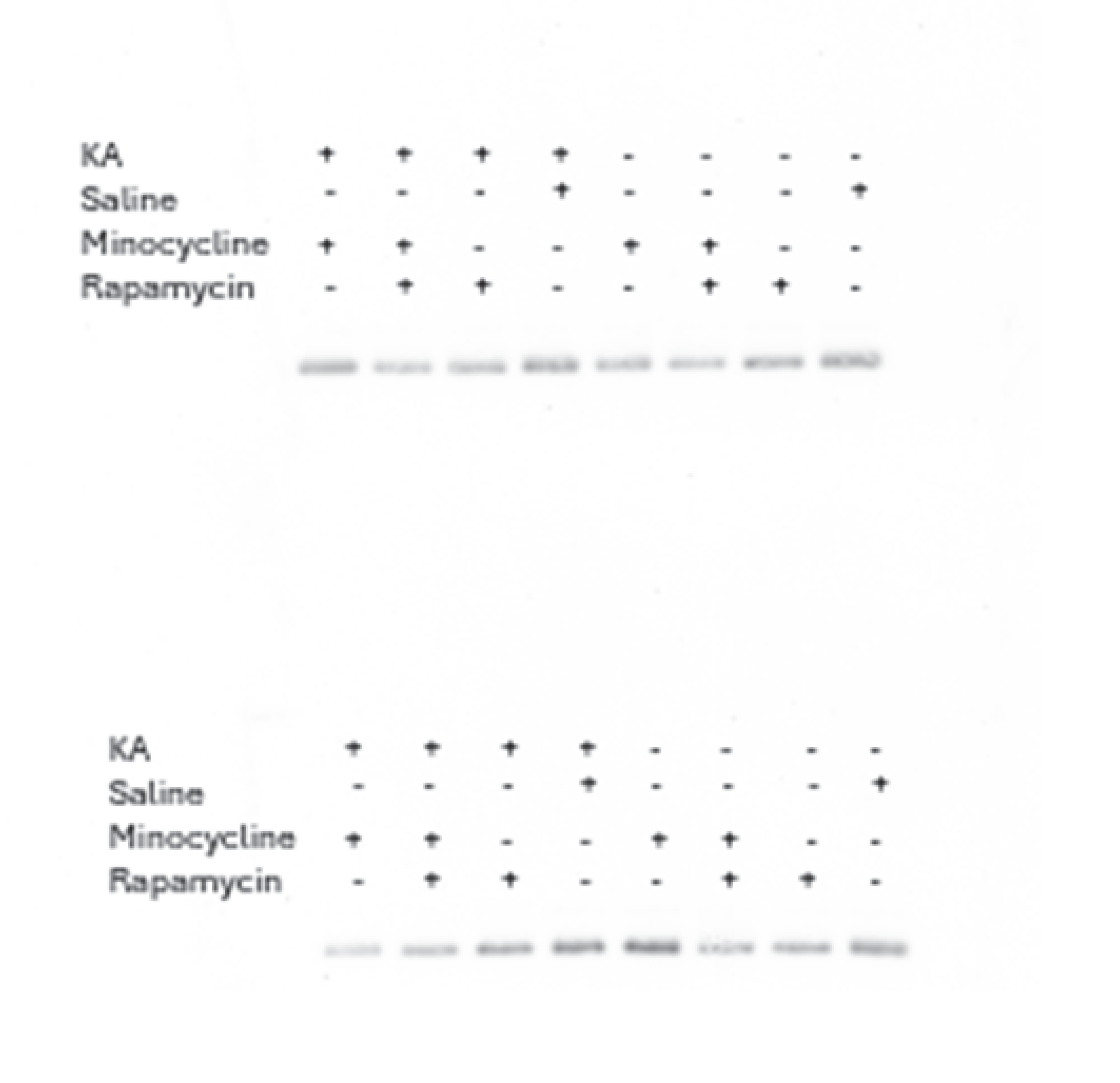

Supplement: Supplementary file 1 [file neurosci-07-00055-s001.zip › Western blots 4-7-26/7_8_S6_bri.tif]
